# The contribution of genetic and environmental influences underlying disordered eating to exposure to weight-conscious peers

**DOI:** 10.1017/S0033291725102948

**Published:** 2026-01-09

**Authors:** Shannon M. O’Connor, S. Alexandra Burt, S. Mason Garrison, Kelly L. Klump

**Affiliations:** 1 Department of Psychology, University of Toledo, Toledo, OH, USA; 2 Department of Psychology, Michigan State University, East Lansing, MI, USA; 3 Department of Psychology, Wake Forest University, Winston-Salem, NC, USA

**Keywords:** behavior genetics, bivariate, Cholesky decomposition, disordered eating, genetic, peers, twin study, weight-conscious

## Abstract

**Background:**

Girls with predispositions for disordered eating (DE) may select into weight-conscious peer groups (i.e. peer groups that emphasize body weight/shape). However, factors driving selection into these peer groups remain unknown, as genetic and/or environmental predisposition to DE may lead girls to select weight-conscious peers. To explore what may drive selection, the present study investigated whether genetic or shared environmental influences underlie associations between DE and exposure to weight-conscious peers and whether effects differ by pubertal status.

**Methods:**

Participants included 833 female twins (ages 8–15) from the Michigan State University Twin Registry. Bivariate twin models were conducted to explore etiologic overlap between DE and exposure to weight-conscious peers. Separate models were run for pre-early pubertal girls and mid-late pubertal girls given past research demonstrates differences in genetic and environmental contributions underlying eating pathology by pubertal status.

**Results:**

During pre-early puberty, shared and non-shared environmental correlations accounted for the overlap between DE and weight-conscious peer group exposure. Furthermore, shared environmental and non-shared environmental influences underlying DE contributed to 33.3% and 20.0% of the individual differences in weight-conscious peer group membership, respectively. In mid-late puberty, the genetic and non-shared environmental correlations accounted for the overlap between DE and weight-conscious peer group exposure. Genetic and non-shared environmental influences underlying DE contributed to 37.5% and 19.4% of the variance in weight-conscious peer group membership, respectively.

**Conclusions:**

While selection effects may exist across development, these effects may be driven by variance in DE due to shared environment in pre-early puberty and genes in mid-late puberty.

Membership in weight-conscious peer groups (i.e. peer groups highly focused on topics such as attractiveness, body weight, body shape, exercise, and dieting; Van Huysse et al., 2016) has been associated with higher rates of body weight concerns, dieting, and body dissatisfaction in both cross-sectional (e.g. Paxton et al, 1999; Taylor et al., 1998; Vander Wal & Thelen, 2000) and longitudinal (e.g. Keel et al, 2013) studies. Prior research has attributed these associations to socialization effects, such that membership in weight-conscious peer groups leads to higher rates of disordered eating (DE) due to being exposed to peers who strongly emphasize dieting, appearance, and thin body weights/shapes. Exposure to these weight-conscious peers may reinforce maladaptive eating and body-related cognition, which exacerbate disordered eating symptoms (e.g. Crandall, 1988). However, peer selection is not random, as individuals may seek out peers based on their own attitudes and beliefs or traits they deem desirable (e.g. Boutwell, Meldrum, & Petkovsek, 2017; Rayner et al., 2013). Given that individuals’ attitudes and behaviors may be genetically or environmentally influenced, an individual’s genetic or environmental predisposition may lead them to select into a particular peer group. Thus, the association between weight-conscious peers and elevated DE could be due to girls with a predisposition for DE selecting into peer groups who share their emphasis on weight, shape, and appearance.

Few studies have examined potential selection effects for DE and weight-conscious peer groups (see summary in O’Connor et al, 2016). However, emerging data suggest that selection effects may contribute to these associations. Using a co-twin control design, O’Connor et al. (2016) demonstrated support for the presence of genetic and/or shared environmental selection effects as opposed to pure socialization effects. These findings were consistent with previous studies that support the presence of selection effects, including a longitudinal study that found that girls (ages 12 to 14) were more likely to stay in friendship groups that were more similar to their own level of body dissatisfaction and bulimic behaviors (Rayner et al., 2013) and a study that found *unselected* college roommates did not become more similar in their bulimic symptoms (suggesting a lack of socialization effects when initial selection has not occurred) (Myer & Waller, 2001). Thus, selection factors may be important in the relationship between exposure to weight-conscious peers and DE; however, it is unclear whether these effects are driven by genetic or environmental factors.

Indeed, one limitation of O’Connor et al. (2016) was that the study design was unable to disentangle the *extent* to which selection was driven by additive genetic (i.e. genetic influences that add across genes) and/or shared environmental (i.e. environmental influences that are shared by reared-together twins and are thus a source of their behavioral similarity) influences. Specifically, while it was clear that genetic and/or shared environmental influences were important, within this model there was no way of estimating the contribution of these sources of influence. Understanding what underlies these selection effects can provide a more comprehensive picture of how peers influence DE and direct future research on what specific genetic or environmental influences lead individuals to select weight-conscious peers.

The present study used a bivariate twin design to explore (1) which etiologic influences account for the overlap between DE and exposure to weight-conscious peers and (2) to what extent genetic and environmental variance in DE contribute to individual differences in exposure to weight-conscious peers. This study used the same measures and largely overlapping sample (65%) as O’Connor et al. (2016), allowing for extension in a sample known to have demonstrated selection effects.

Importantly, O’Connor et al. (2016) did not find differences in selection versus socialization in pre to early (pre-early) pubertal and mid to late (mid-late) pubertal individuals – that is, genetic and shared environmental selection effects were present across adolescence, regardless of pubertal stage. However, this does not rule out the possibility that the influences driving selection effects could change across development. Indeed, prior studies examining developmental differences in genetic/environmental influences on DE symptoms only have shown dramatic differences across puberty (i.e. substantial shared environmental influence in pre-early puberty and significant genetic influences in mid-late puberty; Culbert et al., 2009; Klump et al., 2000; 2007; Klump, McGue, & Iacono, 2003; O’Connor et al., 2020) that may translate into differential etiologic associations with weight-focused peer groups as well. Consequently, we conducted models separately in pre-early pubertal and mid-late pubertal samples to examine this possibility. Notably, past developmental twin studies have highlighted these shifts across a number of DE constructs, including DE assessed broadly (e.g. Culbert et al., 2009; Klump et al., 2007, 2013), binge eating (Klump et al., 2017), and cognitive symptoms of DE (e.g. body dissatisfaction, weight/shape concerns, weight preoccupation; O’Connor et al., 2020). Furthermore, O’Connor et al. (2016) included multiple measures of DE; however, results were largely similar across DE constructs. Within the present paper, we focus on overall DE and include results for each of the DE constructs used in O’Connor et al. (2016) in Supplemental Tables 2–6. Given past work, we hypothesized that shared environmental influences would be more important for the association between weight-conscious peers and DE prior to mid-puberty, whereas genetic influences would contribute to the association in mid-late puberty.

## Methods

### Participants

Participants included 833 female twins ages 8–16 years (M = 11.69, SD = 2.10; 51.6% monozygotic (MZ) or identical twins who share 100% of their segregating genes, 48.4% dizygotic (DZ) or fraternal twins who share approximately 50% of their segregating genes) from the *Twin Study of Mood, Behavior, and Hormones during Puberty* (TSMBH; Klump et al., 2018) within the Michigan State University Twin Registry (Burt & Klump, 2013, 2019; Klump & Burt, 2006). Information on the recruitment, eligibility criteria, and TSMBH sample can be found in Appendix A in the Supplemental Materials.

### Zygosity determination

Zygosity was determined using a well-validated physical similarity questionnaire (Lykken, Bouchard, McGue, & Tellegen, 1990) that demonstrates 95% accuracy when compared with genotyping (Peeters, Van Gestel, Vlietinck, Derom, & Derom, 1998). Both twins, their mother, and two research assistants evaluated the physical similarities independently. Reports were compared and discrepancies were resolved through review of questionnaire data and twin photographs by one of the principal investigators (KLK) or by examination of DNA markers (Burt & Klump, 2019).

### Measures

#### Exposure to weight-conscious peer groups

The same self-report measures used in O’Connor et al. (2016) were used to assess exposure to weight-conscious peer groups (as described below). These questionnaires tap related constructs, as inter-correlations between questionnaires were moderate-to-large (*r*s = .25–.58, mean *r = .*43, SD = 0.12), and previous analyses suggest very similar results across the different peer exposure questionnaires (O’Connor et al, 2016). Thus, the present study used a peer exposure composite score to decrease the number of models fit; however, models using each individual peer exposure questionnaire were included in Supplemental Tables 7–11. More details about the creation of the composite score are provided following the description of the four peer exposure questionnaires below.

The *Perceived Friend Preoccupation with Weight and Dieting Scale* (Schutz, Paxton, & Wertheim, 2002) is a 9-item self-report questionnaire that aims to assess frequency of weight- and dieting-related thoughts and behaviors among their friends. Participants are asked to rate their response on a 5-point Likert scale. Good internal consistency has been suggested in a previous study of adolescent girls ( 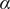 = .87) and in the present sample ( 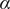 = .86; McDonald’s Ω = .86).

The *Appearance Conversations with Friends* (*Jones, Vigfusdottir, & Lee, 2004*) is a 5-item questionnaire that assesses the frequency of discussions about current and desired body shape with friends. This questionnaire was modified from the original Magazines as a Source of Influence Scale (Levine, Smolak, & Hayden, 1994). This scale has demonstrated good internal consistency in prior studies ( 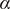 = .85; Jones et al., 2004) and in the present sample ( 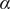 = .89; McDonald’s Ω = .89).

The *Friends as a Source of Influence Scale* (Paxton et al. 1999) is a 5-item questionnaire that assesses how important participants think their friends’ opinions are in influencing their ideas of the ‘perfect’ body, diet products, exercise, and dieting. Participants are asked to rate each item on a 5-point Likert scale. Good internal consistency was found in prior studies ( 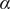 = .87; Paxton et al. 1999), as well as in the present sample ( 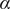 = .83; McDonald’s Ω = .84).

The *Peer Attribution Scale* (Lieberman, Gauvin, Bukowski, & White, 2001) includes 8-item assessing appearance-related attributions from friends on a 6-point Likert scale. The original scale includes items referring to same-sex and opposite-sex friends; however, in the current sample, we used a modified 4-item version (Shroff & Thompson, 2006) that refers to any friend (same- or opposite-sex). This modified scale exhibited good internal consistency in past research ( 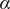 = .85; Shroff &Thompson, 2006) and adequate internal consistency in the present study ( 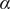 = .79; McDonald’s Ω = .81).

A *Composite Measure* of exposure to weight-conscious peer groups was created by standardizing (via z-scores) each of the four peer questionnaires and computing an average score. While most participants had data for all four peer questionnaires (n = 750, 90%), a minority had data available for only three of the four peer measures (n = 56, 6.7%). The composite score was computed for all individuals with data available for at least three of the four peer measures by taking the average of the available questionnaires. Participants missing two or more peer measures (n = 27, 3.2%) were recoded as missing. Internal consistency of this composite peer exposure score was excellent in the current sample ( 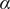 = .91; McDonald’s Ω = .91).

#### Disordered eating

DE was assessed using the *Minnesota Eating Behavior Survey* (MEBS; von Ranson, Klump, Iacono & McGue, 2005; the Minnesota Eating Behavior Survey [MEBS; previously known as the Minnesota Eating Disorder Inventory (M-EDI)] was adapted and reproduced by special permission of Psychological Assessment Resources, Inc., 16204 North Florida Avenue, Lutz, Florida 33549, from the Eating Disorder Inventory [collectively, EDI and EDI-2] by Garner, Olmstead, & Polivy (1983) Copyright 1983 by Psychological Assessment Resources, Inc. Further reproduction of the MEBS is prohibited without prior permission from Psychological Assessment Resources, Inc.), a 30-item questionnaire made up of true/false questions that assesses a range of DE symptoms. This measure was developed for use with children as young as 10 years old; however, past studies have supported its use in girls as young as 8 years old (e.g. Luo, Donnellan, Burt, & Klump, 2016). The present study focused on the MEBS Total Score (i.e. sum score of all 30 items). The MEBS Total Score demonstrates good internal consistency in samples of adolescent girls ( 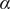 = .86–.87; von Ranson et al., 2005) and within the present sample ( 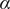 = .84). (We were unable to include McDonald’s omega for MEBS Total Score as low endorsement of some items prevented calculation [i.e. lead to negative inter-item correlations].)

#### Pubertal Development Scale

The Pubertal Development Scale (PDS; Petersen, Crockett, Richards, & Boxer, 1988) is a self-report questionnaire in which participants rate their pubertal development based on the physical signs of puberty (i.e. height spurts, body hair growth, skin changes, breast development, onset of menarche) on a 4-point scale: (1) development has not yet begun; (2) development has barely started; (3) development is definitely underway; and (4) development seems completed. An exception to this 4-point scale is the coding for menses, which is coded dichotomously (absent (1) or present (4)). Similar to previous studies (e.g., Culbert et al., 2009; Klump et al., 2003), the ratings of each physical marker were averaged to obtain an overall PDS score, with higher scores representing more advanced pubertal development. The PDS exhibits good psychometric properties and correlates highly (*r* = .61–.67) with physician ratings of pubertal development (Petersen et al., 1988).

Given past research demonstrated differences in genetic and environmental contributions underlying eating pathology in pre-early puberty vs mid-late puberty and adulthood (Culbert et al., 2009; Klump et al., 2007, 2013, 2017; O’Connor et al., 2020), models were conducted separately in these two groups using a cutoff established in past research (i.e. PDS score < 2.5 = pre-early puberty; PDS score ≥ 2.5 = mid-late puberty). Sixty-six percent (275/419 pairs) of twins comprised the pre-early pubertal group, whereas the remaining (144/419 pairs) comprised the mid-late pubertal group. Twin pairs discordant on pubertal status were excluded from analyses as they could not be included in both pubertal groups (n = 122 twins). These twins were not included in our reported sample size (N = 833). Notably, discordant twins did not differ in their level of DE (*t(*968) = .377, *p* = .35) or exposure to weight focused peers (*t(*941) = −.132, *p* = .45) compared to twins concordant on pubertal status with their co-twin.

#### Covariates: body mass index (BMI) and age

Given demonstrated associations between body mass index (BMI) within peer groups (Trogdon, Nonnemaker, & Pais, 2008), BMI was included as a covariate to ensure that the associations between peer group and DE were independent of BMI. BMI was calculated (weight in kg/ height in m^2^) using height and weight measured with a wall-mounted ruler and digital scale.

A variety of ages were present in each pubertal group. Age was regressed out of the disordered eating and exposure to weight-conscious peer composite scores, and the unstandardized residuals were used to ensure differences in age were not driving effects.

#### Statistical analyses

Twin intraclass correlations (e.g. correlation between the MEBS total score of Twin 1 and the MEBS total score of Twin 2) and cross-twin, cross-trait correlations (e.g. correlation between the MEBS total score of Twin 1 and the composite weight-conscious peer group score of Twin 2) were calculated to provide initial indications of genetic and environmental influences.

##### Bivariate twin models

Bivariate twin models were fit to raw data using the full maximum likelihood option in Mx (Neale et al., 1995). The full maximum likelihood option assumes missing data are missing at random and allows for less biased and more consistent estimates than pairwise or listwise deletion. Only three twin pairs had one missing co-twin; the full maximum likelihood option allowed for retaining the data of the participating co-twin within the dataset. The current study aimed to determine what etiologic influences account for overlap between DE and weight-conscious peer group exposure. Bivariate twin models were used to calculate genetic (r_a_), shared environment (r_c_), and non-shared environment (r_e_) correlations between DE and exposure to weight-conscious peer groups.

**Figure 1.**
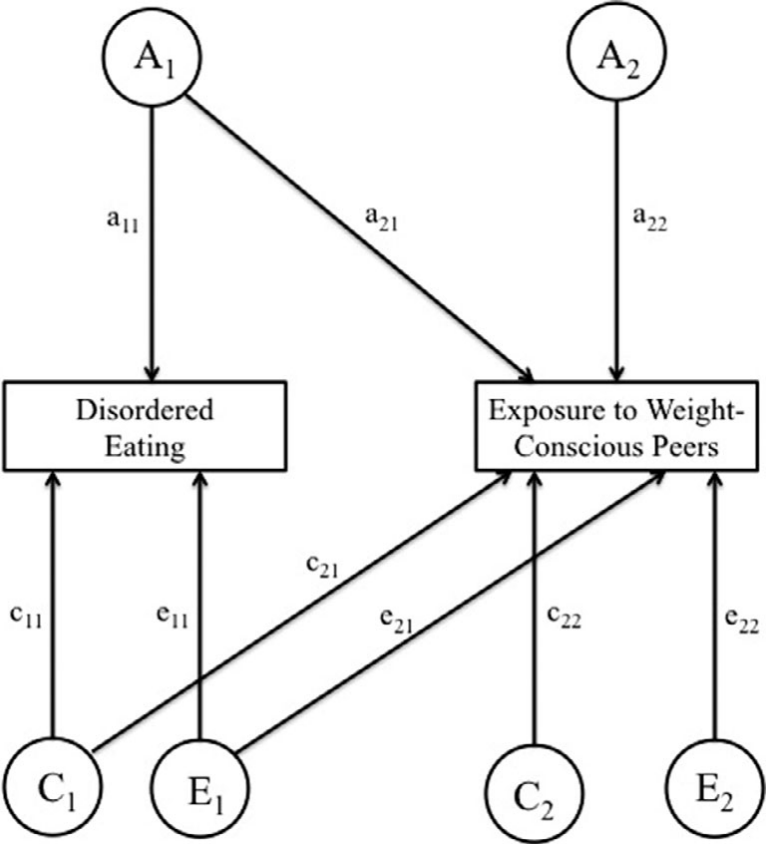
Path diagram of Cholesky ACE model for exposure to weight-conscious peers and disordered eating. Variance in each attribute is assumed to be determined by the additive combination of the three latent factors: additive genetic (A), shared environmental (C), and non-shared environmental (E) effects. The additive genetic, shared environmental, and non-shared environmental variances in the exposure to weight-conscious peers composite score are partitioned into those components attributable to the genetic and environmental effects disordered eating (a_21_, c_21_, e_21_) and the residual components that are independent of the genetic and environmental effects of disordered eating (a_22_, c_22_, e_22_).

Next, we explored the extent to which the variance in exposure to weight-conscious peers may be accounted for by the variance in DE using Cholesky decomposition models. Cholesky decomposition is a multivariate technique based on the principles of factor analysis that provides estimates of additive genetic (A), shared environmental (C), and nonshared environmental (E) contributions to variance in, and to covariance between, exposure to weight-conscious peers and DE (see Figure 1). Importantly, while the ordering of variables within the Cholesky model does not affect the model fit, ordering DE first (as shown on the left-hand side of Figure 1) allows the variance in exposure to weight-conscious peers to be decomposed into components attributable to the genetic and environmental effects on DE (depicted as a_21_,c_21_, and e_21_) and the residual components that are independent of the genetic and environmental variance in DE (depicted as a_22_,c_22_, and e_22_). The variance in DE is not decomposed into components attributable to exposure to weight-conscious peers and those solely DE. Indeed, variance in DE is only decomposed into the total variance due to genetic, shared environment, and non-shared environmental influences (depicted as a_11_, c_11_, and e_11_ in Figure 1). The following statistics were derived from the estimated parameters: the heritability of DE (h_1_^2^); the heritability of exposure to weight-conscious peer groups, both overall (h_2_^2^) as well as decomposed into a portion attributable to genetic effects on DE (h_a_^2^) and a residual component (h_r_^2^); the influence of shared and non-shared environment underlying DE (c_1_^2^ and e_1_^2^, respectively); the influence of shared and non-shared environment underlying exposure to weight-conscious peers groups overall (c_2_^2^ and e_2_^2^, respectively) as well as decomposed into a portion attributable to shared and non-shared environmental effects on DE (c_a_^2^ and e_a_^2^, respectively) and residual components (c_r_^2^ and e_r_^2^, respectively).

Four models were fit to the data: the full ACE, CE, AE, and E models. Model fit was compared by taking the difference in minus twice the log-likelihood (−2lnL) between the full and nested models, which is chi-squared distributed under the null hypothesis implied by the reduced model. Large (statistically significant) differences led to a rejection of the nested model. Additionally, low estimates on Akaike’s information criteria (AIC; Akaike, 1987), Bayesian information criterion (BIC; Raftery, 1995), sample-size adjusted BIC (SABIC; Sclove, 1987), and deviance information criterion (DIC; Spiegelhalter, Best, Carlin, & Van Der Linde, 2002) were also used to select the best fitting model, as these indices differentially weigh model fit versus model parsimony.

## Results

### Descriptive data

A range of DE and weight-conscious peer composite scores were present in our sample (see Table 1). Given our samples age range (ages 8–16; M = 11.69, SD = 2.10), the proportion of our sample that scored above the clinical cut-off (score = 15.55) for the MEBS Total Score was within an expected range (n = 23, 2.7%; von Ranson et al., 2005). Additionally, 15.1% of twins scored >1 SD above the mean on the composite score for exposure to weight-conscious peers. As expected, the mid-late pubertal group reported higher levels of DE and more weight-conscious peers than the pre-early pubertal sample (DE: *t*(507.334) = −3.12, *p* < .001, Cohen’s *d* = .23; exposure to weight-conscious peers: *t*(506.21) = −10.24, *p* < .001, Cohen’s *d* = .78).

**Table 1.** Descriptive statistics

Variables	Total sample	Categorical puberty categories	
		Pre-/early pubertal group	Mid-/late pubertal group
**Sample sizes**			
MZ twins	430	286	144
DZ twins	403	260	143
***Total***	***833***	***546***	***287***
**Age**			
Range	8.02–16.01	8.02–14.45	8.75–16.01
Mean (SD)	11.69 (2.10)	10.47 (1.26)	14.02 (1.24)
**Body mass index**			
Range	10.70–39.67	10.70–37.42	15.48–39.67
Mean (SD)	19.43 (4.39)	17.91 (3.50)	22.33(4.47)
**PDS scores**			
Range	1.00–4.00	1.00–2.40	2.60–4.00
Mean (SD)	2.18 (.91)	1.59 (.42)	3.31 (.38)
**MEBS total score**			
Range (max score = 30)	0–19	0–19	0–19
Mean (SD)	4.09 (4.11)	3.75 (3.84)	4.73 (4.52)
Alphas	.84	.83	.85
Skewness	1.29	1.41	1.07
Kurtosis	1.17	1.72	0.36
**Composite peer exposure**			
Range (possible: 23–119)	23–78	23–78	23–78
Mean (SD)	36.61 (12.42)	33.39 (10.69)	42.77 (13.18)
Alphas	.91	.90	.90
Skewness	.81	1.18	.33
Kurtosis	.02	1.28	−.68

*Note.* MEBS, Minnesota Eating Behavior Survey; PDS, Pubertal Development Scale; SD, standard deviation. Sample sizes indicate the number of twins (not the number of twin pairs).

### Pearson correlations and twin correlations

As expected, significant phenotypic associations were observed between exposure to weight-conscious peers and DE (*r*s = .51–.59, *p* < .001; see Table 2). Twin intraclass correlations provide an initial indication of the degree to which these associations are due to genetic versus environmental factors. Within the pre-early pubertal sample, shared environmental and non-shared environmental effects were suggested for DE and exposure to weight-conscious peers, as the MZ and DZ correlations were not significantly different from one another (indicating shared environmental effects) and less than 1.0 (indicating non-shared environmental effects). Within the mid-late pubertal sample, genetic and non-shared environmental effects were suggested for both DE and exposure to weight-conscious peers, as the MZ correlations were greater than DZ correlations (indicating genetic effects), and the MZ correlation was less than 1.0 (indicating non-shared environmental effects). Cross-twin, cross-trait correlations also indicated shared environmental influences underlying the association between DE and weight-conscious peers in pre-puberty, as correlations were similar in MZ and DZ twin pairs. However, genetic influences were suggested to underlie the association between DE symptoms and exposure to weight-conscious peers in the mid-late pubertal sample.

**Table 2.** Phenotypic, intraclass, and cross-twin, cross-trait correlations

	Phenotypic	Intraclass correlations						Cross-twin, cross trait					
	Correlation	MZ	DZ	Z	p	q	q interpretation	MZ	DZ	Z	p	q	q interpretation
*Pre-early pubertal*													
Disordered eating	.51***	.44***	.42***	.33	.37	.03	No effect	.24***	.27***	−.37	.36	.03	No effect
Peer exposure		.46***	.48***	−.31	.38	.03	No effect						
*Mid-late pubertal*													
Disordered eating	.59***	.66***	.32***	3.95***	<.001	.46	Medium effect	.40***	.20*	1.86*	.03	.22	Small effect
Peer exposure		.60***	.35***	2.60**	<.01	.33	Medium effect						

*Note.* Disordered Eating, Total Score on the Minnesota Eating Behavior Survey; DZ, dizygotic; MZ, monozygotic; Peer Exposure, composite score on 4 peer measures; Phenotypic correlation, Pearson’s correlation between the disordered eating symptom and the composite peer exposure score for each individual; q, effect size that indexes the magnitude of the difference between MZ and DZ twin correlations (q values of .10 are considered small, .30 is considered medium, and .50 is considered large); Z, Z test of equality that tests for differences between the MZ and DZ twin correlations.

* p < .05.

** p < .01.

*** p < .001 one-tailed. The correlation is significantly different from zero.

### Bivariate twin models

Table 3 provides comparisons of the four bivariate models (i.e. ACE, CE, AE, and E models). The CE model provided the best fit to the data in the pre-early pubertal sample, and the AE model was best fitting in the mid-late pubertal sample; these models had the lowest AIC, BIC, SABIC, and DIC values and as demonstrated non-significant changes in chi-square values between the full model (i.e. ACE) and best-fitting submodel.

**Table 3.** Comparison of nested bivariate Cholesky decomposition models

Model	*χ^2^* (*df*)	*χ^2^* diff (*df*)^a^	p	AIC	BIC	SABIC	DIC		*χ^2^* (*df*)	*χ^2^* diff (*df*)^a^	p	AIC	BIC	SABIC	DIC
Pre-early pubertal sample (n = 275 twin pairs)									Mid-late pubertal sample (n = 144 twin pairs)						
ACE	2304.27 (1068)			168.27	−1847.22	−154.01	−865.79		1335.04 (548)			239.04	−692.30	174.68	−188.72
AE	2314.01 (1071)	9.74 (3)	.02	172.01	−1850.78	−152.81	−866.59		**1335.20 (551)**	**.16 (3)**	**.98**	**233.20**	**−699.66**	**172.07**	**−193.33**
CE	**2307.67 (1071)**	**3.4 (3)**	**.33**	**165.67**	**−1853.95**	**−155.98**	**−869.76**		1346.93 (551)	11.89 (3)	.01	244.93	−693.80	177.93	−187.46
E	2407.67 (1074)	103.4 (6)	<.01	259.67	−1812.37	−109.65	−825.43		1394.09 (554)	59.05 (6)	<.01	286.09	−677.66	198.81	−168.57

*Note. χ^2^* diff = chi-squared difference test; AIC, Akaike’s information criterion; BIC, Bayesian information criterion; DIC, deviance information criterion; SABIC, Sample-size adjusted Bayesian information criterion. The best fitting model is indicated in boldface.

a AE, CE, and E models are compared with the full ACE model for the *χ^2^* diff test.

Significant bivariate correlations were calculated in each of the best fitting models (see Table 4). In pre-early puberty, DE and exposure to weight-conscious peers had an r_c_ = 0.58 and r_e_ = 0.45, indicating that the proportion of the shared and non-shared environmental variance of one trait that was shared with the other was 0.34 (=0.58*0.58) and 0.20 (=0.45*0.45), respectively. In mid-late puberty, DE and exposure to weight-conscious peers had an r_a_ = .61 and r_e_ = .44, indicating that the proportion of the genetic and non-shared environmental variance of one trait that was shared with the other was 0.37 (=0.61*0.61) and 0.19 (=0.44*0.44), respectively.

**Table 4.** Parameter estimates for bivariate model for disordered eating symptoms and exposure to weight-conscious peers composite score (pre-early puberty: 275 twin pairs; mid-late puberty: 144 twin pairs)

	Pre-pubertal sample		Pubertal sample	
	Full model: ACE	Best fitting: CE	Full Model: ACE	Best fitting: AE
**Bivariate correlations**				
Additive genetic (r_a_)	−1.00 (−1.00, 1.00)	-	**.66 (.25, 1.00)**	**.61 (.41, .76)**
Shared environmental (r_c_)	**.86 (.43, 1.00)**	**.58 (.41, .72)**	−1.00 (−1.00, 1.00)	-
Non-shared environmental (r_e_)	**.52 (.39, .62)**	**.45 (.35, .54)**	**.43 (.23, .59)**	**.44 (.25, .60)**
**Cholesky decomposition models**				
Heritability estimate				
MEBS total score				
Total (h^2^)	.09 (.00, .42)	-	**.52 (.19, .65)**	**.52 (.36, .65)**
Peer composite score				
Total (h^2^)	.09 (.00, .42)	-	**.58 (.15 .75)**	**.64 (.48, .76)**
Attributable (h^2^_a_)	.09 (.00, .39)	-	**.25 (.02, .67)**	**.24 (.09, .40)**
Residual (h^2^_r_)	<.01 (.00, .41)	-	.33 (.00, .54)	**.41 (.26, .54)**
% Attributable	100%	-	43.1%	37.5%
Shared environmentality estimate				
MEBS total score				
Total (c^2^)	**.34 (.06, .48)**	**.41 (.30, .50)**	<.01 (.00, .28)	-
Peer composite score				
Total (c^2^)	**.39 (.12, .54)**	**.45 (.35, .54)**	.06 (.00, .39)	-
Attributable (c^2^_a_)	**.28 (.05, .51)**	**.15 (.07, .26)**	.06 (.00, .39)	-
Residual (c^2^_r_)	.10 (.00, .34)	**.30 (.22, .39)**	<.01 (.00, .35)	-
% Attributable	71.8%	33.3%	100%	-
Non-shared environmentality estimate				
MEBS total score				
Total (e^2^)	**.57 (.46, .68)**	**.59 (.50, .70)**	**.48 (.35, .64)**	**.48 (.35, .64)**
Peer composite score				
Total (e^2^)	**.52 (.41, .63)**	**.55 (.46, .65)**	**.36 (.25, .54)**	**.36 (.24, .52)**
Attributable (e^2^_a_)	**.14 (.07, .21)**	**.11 (.06, .18)**	**.07 (.02, .16)**	**.07 (.02, .16)**
Residual (e^2^_r_)	**.38 (.29, .48)**	**.43 (.36, .52)**	**.29 (.20, .44)**	**.29 (.20, .42)**
% Attributable	26.9%	20.0%	19.4%	19.4%

*Note.* % Attributable, the percentage of variance attributable to MEBS Total Score out of the total variance underlying the peer composite score; c^2^, shared environmentality; c^2^_a_, shared environmentality of the peer composite score that is attributable to common shared environmental effects of MEBS Total Score; c^2^_r_, shared environmentality of the peer composite score that is independent of shared environmental effects of MEBS Total Score; e^2^, non-shared environmentality; e^2^_a_, non-shared environmentality of the peer composite score that is attributable to common non-shared environmental effects of MEBS Total Score; e^2^_r_, non-shared environmentality of the peer composite score that is independent of non-shared environmental effects of MEBS Total Score; h^2^, total heritability; h^2^_a_, heritability of the peer composite score that is attributable to genetic effects of MEBS Total Score; h^2^_r_, heritability of peer composite score that is independent of genetic effects of MEBS Total Score; MEBS, Minnesota Eating Behaviors Survey.

Estimates are followed by 95% confidence intervals in parentheses. Confidence intervals that do not overlap with zero indicate statistical significance (bolded) at p <.05.

Parameter estimates from the full model (ACE) and best-fitting models from the Cholesky decomposition models are also shown in Table 4. Notably, the full (ACE) model indicated non-significant estimates of A for the pre-early pubertal sample and non-significant estimates of C for the mid-late pubertal sample, confirming the appropriate selection of the CE and AE models as best-fitting for the pre-early pubertal and mid-late pubertal samples, respectively. Findings from the best-fitting models were consistent with prior twin studies exploring DE (e.g., Klump et al., 2007, 2010), with significant shared environmental influences in the pre-early pubertal sample (c^2^ = .41) and significant genetic influences in the mid-late pubertal sample (h^2^ = .52). Non-shared environmental influences were prominent for DE in both pre-early puberty (e^2^ = .59) and mid-late puberty (e^2^ = .48). Similarly, shared environmental influences (c^2^ = .45) and non-shared environmental influences (e^2^ = .55) were significant for exposure to weight-conscious peers in pre-early puberty, whereas genetic influence (h^2^ = .64) and non-shared environmental influences (e^2^ = .36) were significant in mid-late puberty. No study to date has explored the etiologic influences underlying exposure to weight-conscious peers, and interestingly, differences across puberty mirror existing and current results underlying DE.

While genetic and environmental influences on DE were consistent with prior studies, the novelty of the Cholesky decomposition models is the ability to explore what proportion of genetic and shared environmental variance in exposure to weight-conscious peers is attributable to genetic and shared environmental variance in DE. Within the pre-early pubertal sample, bivariate estimates suggest that the relationship between DE and exposure to weight-conscious peers is primarily mediated through shared environmental and non-shared environmental effects. Specifically, 33% of variance in exposure to weight-conscious peers was due to shared environmental influences attributable to DE, and 20% of variance in exposure to weight-conscious peers was due to non-shared environmental factors attributable to DE (see Table 4). Nonetheless, results also suggest that the bulk of environmental variance in weight-conscious peer groups is independent of DE, as residual estimates indicated that 67% of shared environmental influences and 80% of non-shared environmental influences underlying exposure to weight-conscious peers is unique/independent of DE (as calculated by subtracting the percent attributable from 100%).

Within the pubertal sample, bivariate estimates suggest that the association between DE and exposure to weight-conscious peers is primarily mediated through genetic effects and non-shared environmental effects. Specifically, 38% of genetic influences and 19% of non-shared environmental influences underlying exposure to weight-conscious peers is attributable to DE (see Table 4). Thus, 62% of genetic influences and 81% of non-shared environmental influences underlying exposure to weight-conscious peers is independent of variance in DE.

## Discussion

Individual differences in DE may at least partial account for exposure to weight-conscious peers. Indeed, our findings suggest that some of the same genetic/environmental influences that underlie an individual’s vulnerability toward DE may also lead to exposure to weight-conscious peers. Approximately one third of the genetic/shared environmental influences underlying weight-conscious peer exposure is attributable to the same etiologic factors underlying DE. Importantly, these shared underlying influences differ across development with shared and non-shared environmental influences underlying the overlap between DE and weight-conscious peers in pre-early puberty and genetic and non-shared environmental influences underlying the overlap between DE and weight-conscious peers in mid-late puberty. Interestingly, the concept that an individual may select into a particular environment based on genetic propensities is known as active gene–environment correlation (‘active rGE’). To our knowledge, despite the extensive literature exploring the influence of peers on DE, the present study is the first to demonstrate the possibility of active rGE within this relationship.

Importantly, our findings mirror developmental shifts found in past twin studies. Indeed, the etiologic differences underlying the relationship between DE and exposure to weight-conscious peers across puberty are consistent with prior developmental twin studies that demonstrate identical shifts for disordered eating (Klump et al, 2007, 2010, 2017; O’Connor et al., 2020). Prior work that has identified environmental risk factors for DE during pre-/early puberty may point to factors that may drive selection of weight-conscious peers during this developmental period. For instance, prior twin family studies have highlighted that parents likely influence offspring via the shared environment during pre-early puberty (O’Connor et al., 2019, 2022). Thus, girls who observe parental DE could select into peer groups who also exhibit these behaviors. The increase in genetic influence that has been demonstrated at mid-late puberty into adulthood may be driven by the influx of ovarian hormones, as twin and animal studies indicate that at least part of the female-specific risk for DE is due to genetic factors associated with estrogen activation at puberty (e.g. Klump, 2013). This activation of genetic risk during mid-puberty could then drive exposure to weight-conscious peers. Interestingly, progesterone (another ovarian hormone that increases at puberty) has demonstrated moderating effects of the association between perceived social pressure to conform to the thin ideal and body image concerns (Forney et al., 2019), suggesting that hormone changes at puberty may directly impact responses to social environments or indirectly through activating genetic vulnerabilities to DE (e.g. Klump, 2013). Future longitudinal twin studies with ratings of the parental modeling of DE and/or other factors that may be hypothesized as drivers of selection are needed to further explore how effects may shift over development.

It is also possible that a risk factor for DE could influence exposure to weight-conscious peers across pubertal development but be driven by different etiologic influences in pre-early vs mid-late puberty. For instance, perfectionism exhibits significant associations with a range of eating disorder symptoms (e.g. fasting, binge behaviors) and diagnoses (Forbush, Heatherton & Keel, 2007) and could drive selection via shared environmental influences prior to puberty (e.g. girls raised in an environment with excessively high standards may select peers who are also self-critical and have high standards). Perfectionistic peers could translate their high standards into an emphasis on society’s notion of the ‘ideal’ body and thus, lead to an increased rate of DE symptoms within the group. Alternatively, perfectionism could also drive genetic selection effects following the onset of puberty (e.g. girls who are genetically predisposed to perfectionistic qualities may select into peer groups with other perfectionistic peers). Future studies are needed to better understand what specific risk factors drive selection effects and how the influences of these factors may differ across pubertal development.

Notably, our interpretation of these results has been through the lens of selection given past studies that have highlighted the likely influence of this process. Within this context, genetic and environmental influences underlying DE drive exposure to weight-conscious peers. However, the design of the present study does not necessarily explore selection versus socialization. Our study findings simply highlight that some of the same genetic/environmental influences underlying DE underlie exposure to weight-conscious peers. Another interpretation could be through the lens of socialization, such that shared genetic/environmental factors contribute to who is suspectable to weight/shape socialization. Thus, interpretation of findings relies on integrating current findings with findings from other study designs that more directly explore socialization versus selection.

While this study has many strengths (e.g. exploration of effects across development, modeling of both genetic and environmental influences), it is not without limitations. First, the study used a non-clinical sample and thus, the generalizability of our findings to a clinical population is unknown. Indeed, it is possible that genetic architecture differs by clinical and non-clinical case (Bulik, Sullivan, Wade, & Kendler, 2000). However, DE is a precursor to full clinical eating disorders (Killen, et al., 1996; Prnjak et al., 2021; Stice & Shaw, 2002), estimates of heritability are similar for many disordered eating symptoms compared to clinical eating disorders (i.e. heritabilities >50%; significant influence of additive genetic and non-shared environment across symptoms and diagnoses during adulthood; Bulik, Sullivan, & Kendler, 1998, 2003; Bulik et al., 2006, 2010; Keski-Rahkonen et al., 2005; Klump et al., 2001; Klump, McGue, & Iacono, 2003; Munn et al., 2010; Reichborn-Kjennerud et al., 2003; Rutherford et al., 1993; Slof-Op’t Landt et al., 2008; Wade, Bulik, Neale, & Kendler, 2000; Wade et al., 1999; Wade, Wilkinson, & Ben-Tovim, 2003), and past studies have indicated that DE is best represented on a dimension with clinical eating disorders (Luo et al., 2016), suggesting our findings may generalize to a clinical sample. Second, the cross-sectional nature of our data limits our ability to address causality (i.e. whether exposure to weight-conscious peers leads to DE or DE leads to selection of weight-conscious peers). Third, we were limited to self-report assessments of DE and perceived importance of weight/shape within peer groups, which could inflate associations between exposure to weight-conscious peer groups and DE due to shared method variance (e.g. similarities in response styles may lead to stronger associations between exposure and outcome variables; Podsakoff et al., 2003). Future studies should use additional informants to assess the value of weight/shape topics within a shared peer group. Finally, the mid-late pubertal group may have been under-powered to detect low amounts of shared environmental influence. However, our results demonstrating the importance of additive genetic and non-shared environment during mid-late puberty are consistent with past work that invariably show the importance of additive genetic and non-shared environment and lack of importance of shared environment from mid-late puberty into adulthood (e.g. Bulik, Sullivan, & Kendler, 1998, 2003; Bulik et al., 2010; Klump, McGue, & Iacono, 2003; Klump et al., 2017; Munn et al., 2010; O’Connor et al., 2020; Slof-Op’t Landt et al., 2008; Wade, Bulik, Neale, & Kendler, 2000; Wade et al., 1999; Wade et al., 2003).

Despite these limitations, the present study adds to the existing literature exploring the role of peers in the development of DE. Our findings highlight that vulnerabilities to DE may contribute to exposure to weight-conscious peers, a potential risky environment for individuals with vulnerabilities to DE. The factors driving exposure appear to shift across pubertal development, and future research is needed to elucidate specific environmental and genetic factors that may be driving this relationship.

## Supporting information

10.1017/S0033291725102948.sm001 O’Connor et al. supplementary material O’Connor et al. supplementary material

## Supplementary material

The supplementary material for this article can be found at http://doi.org/10.1017/S0033291725102948.

## References

[r1] Akaike, H. (1987). Factor analysis and AIC. Psychometrika, 52, 317–332.

[r2] Boutwell, B. B., Meldrum, R. C., & Petkovsek, M. A. (2017). General intelligence in friendship selection: A study of preadolescent best friend dyads. Intelligence, 64, 30–35. 10.1016/j.intell.2017.07.002.

[r3] Bulik, C. M., Sullivan, P. F., & Kendler, K. S. (1998). Heritability of binge-eating and broadly defined bulimia nervosa. Biological Psychiatry, 44(12), 1210–1218. 10.1016/s0006-3223(98)00280-7. 9861464

[r4] Bulik, C. M., Sullivan, P. F., & Kendler, K. S. (2003). Genetic and environmental contributions to obesity and binge eating. International Journal of Eating Disorders, 33(3), 293–298. 10.1002/eat.10140. 12655626

[r5] Bulik, C. M., Sullivan, P. F., Tozzi, F., Furberg, H., Lichtenstein, P., & Pedersen, N. L. (2006). Prevalence, heritability, and prospective risk factors for anorexia nervosa. Archives of General Psychiatry, 63(3), 305–312. 10.1001/archpsyc.63.3.305. 16520436

[r6] Bulik, C. M., Sullivan, P. F., Wade, T. D., & Kendler, K. S. (2000). Twin studies of eating disorders: A review. International Journal of Eating Disorders, 27(1), 1–20. 10.1002/(sici)1098-108x(200001)27:1<1::aid-eat1>3.0.co;2-q. 10590444

[r7] Bulik, C. M., Thornton, L. M., Root, T. L., Pisetsky, E. M., Lichtenstein, P., & Pedersen, N. L. (2010). Understanding the relation between anorexia nervosa and bulimia nervosa in a Swedish National Twin Sample. Biological Psychiatry, 67(1), 71–77. 19828139 10.1016/j.biopsych.2009.08.010 PMC2851013

[r8] Burt, S. A., & Klump, K. L. (2013). The Michigan State University twin registry (MSUTR): An update. Twin Research and Human Genetics, 1(1), 1–7. 10.1017/thg.2012.87. PMC5800311 23101567

[r9] Burt, S. A., & Klump, K. L. (2019). The Michigan State University twin registry (MSUTR): 15 years of twin and family research. Twin Research and Human Genetics, 22, 741–745. 10.1017/thg.2019.57. 31466551 PMC7083329

[r10] Crandall, C. S. (1988). Social contagion of binge eating. Journal of Personality and Social Psychology, 55(4), 588–598. 10.1037/0022-3514.55.4.588. 3193348

[r11] Culbert, K. M., Burt, S. A., McGue, M., Iacono, W. G., & Klump, K. L. (2009). Puberty and the genetic diathesis of disordered eating attitudes and behaviors. Journal of Abnormal Psychology, 118(4), 788–796. 10.1037/a0017207. 19899848 PMC2782672

[r12] Forbush, K., Heatherton, T. F., & Keel, P. K. (2007). Relationships between perfectionism and specific disordered eating behaviors. International Journal of Eating Disorders, 40, 37–41. 10.1002/eat.20310. 16958125

[r13] Forney, K. J., Keel, P. K., O’Connor, S. M., Sisk, C., Burt, S. A., & Klump, K. L. (2019). Interaction of hormonal and social environments in understanding body image concerns in adolescent girls. Journal of Psychiatric Research, 109, 178–184. 10.1016/j.jpsychires.2018.12.008. 30553150 PMC6317862

[r65] Garner, D. M., Olmstead, M. P., & Polivy, J. (1983). Development and validation of a multidimensional eating disorder inventory for anorexia nervosa and bulimia. International journal of eating disorders, 2(2), 15–34.

[r15] Jones, D. C., Vigfusdottir, T. H., & Lee, Y. (2004). Body image and the appearance culture among adolescent girls and boys: An examination of friend conversations, peer criticism, appearance magazines, and the internalization of appearance ideals. Journal of Adolescent Research, 19(3), 323–339. 10.1177/0743558403258847.

[r16] Keel, P. K., Forney, K. J., Brown, T. A., & Heatherton, T. F. (2013). Influence of college peers on disordered eating in women and men at 10- year follow-up. Journal of Abnormal Psychology, 122(1), 105–110. 10.1037/a0030081. 23025666 PMC3848030

[r17] Keski-Rahkonen, A., Bulik, C. M., Neale, B. M., Rose, R. J., Rissanen, A., & Kaprio, J. (2005). Body dissatisfaction and drive for thinness in young adult twins. International Journal of Eating Disorders, 37(3), 188–199. 10.1002/eat.20138. 15822080

[r18] Killen, J. D., Taylor, C. B., Hayward, C., Haydel, K. F., Wilson, D. M., Hammer, L., Kraemer, H., Blair-Greiner, A., & Strachowski, D. (1996). Weight concerns influence the development of eating disorders: A 4-year prospective study. Journal of Consulting and Clinical Psychology, 64(5), 936–940. 10.1037//0022-006x.64.5.936. 8916622

[r19] Klump, K. L. (2013). Puberty as a critical risk period for eating disorders: A review of human and animal studies. Hormones and Behavior, 64(2), 399–410. 10.1016/j.yhbeh.2013.02.019. 23998681 PMC3761220

[r20] Klump, K. L., & Burt, S. A. (2006). The Michigan State University twin registry (MSUTR): Genetic, environmental and neurobiological influences on behavior across development. Twin Research and Human Genetics, 9(6), 971–977. 10.1375/183242706779462868. 17254439

[r21] Klump, K. L., Burt, S. A., McGue, M., & Iacono, W. G. (2007). Changes in genetic and environmental influences on disordered eating across adolescence: A longitudinal twin study. Archives in General Psychiatry, 64(12), 1409–1415. 10.1001/archpsyc.64.12.1409. 18056549

[r22] Klump, K. L., Burt, S. A., Spanos, A., McGue, M., Iacono, W. G., & Wade, T. M. (2010). Age differences in genetic and environmental influences on weight and shape concerns. International Journal of Eating Disorders, 43, 679–688. 10.1002/eat.20772. 19950189 PMC2891330

[r23] Klump, K. L., Culbert, K. M., O’Connor, S., Fowler, N., & Burt, S. A. (2017). The significant effects of puberty on the genetic diathesis of binge eating in girls. International Journal of Eating Disorders, 50(8), 984–989. 10.1002/eat.22727. 28560852 PMC5538919

[r24] Klump, K. L., Fowler, N., Mayhall, L., Sisk, C. L., Culbert, K. M., & Burt, S. A. (2018). Estrogen moderates genetic influences on binge eating during puberty: Disruption of normative processes? Journal of Abnormal Psychology, 127(5), 458. 10.1037/abn0000352. 29927265 PMC6060616

[r25] Klump, K. L., Keel, P. K., Burt, S. A., Racine, S. E., Neale, M. C., Sisk, C. L., & Boker, S. (2013). Ovarian hormones and emotional eating associations across the menstrual cycle: An examination of the potential moderating effects of body mass index and dietary restraint. International Journal of Eating Disorders, 46(3), 256–263. 10.1002/eat.22084. 23315657 PMC3600109

[r26] Klump, K. L., McGue, M., & Iacono, W. G. (2000). Age differences in genetic and environmental influences on eating attitudes and behaviors in preadolescent and adolescent female twins. Journal of Abnormal Psychology, 109(2), 239–251. 10895562

[r27] Klump, K. L., McGue, M., & Iacono, W. G. (2003). Differential heritability of eating attitudes and behaviors in prepubertal versus pubertal twins. International Journal of Eating Disorders, 33(3), 287–292. 10.1002/eat.10151. 12655625

[r28] Klump, K. L., Miller, K. B., Keel, P. K., McGue, M., & Iacono, W. G. (2001). Genetic and environmental influences on anorexia nervosa syndromes in a population based twin sample. Psychological Medicine, 31, 737–740. 10.1017/s0033291701003725. 11352375

[r29] Levine, M. P., Smolak, L., & Hayden, H. (1994). The relation of sociocultural factors to eating attitudes and behaviors among middle school girls. Journal of Early Adolescence, 14(4), 471–490. 10.1177/0272431694014004004.

[r30] Lieberman, M., Gauvin, L., Bukowski, W. M., & White, D. R. (2001). Interpersonal influence and disordered eating behaviors in adolescent girls: The role of peer modeling, social reinforcement and body-related teasing. Eating Behaviors, 2, 215–236. 10.1016/s1471-0153(01)00030-7. 15001032

[r31] Luo, X., Donnellan, M. B., Burt, S. A., & Klump, K. L. (2016). The dimensional nature of eating pathology: Evidence from a direct comparison of categorical, dimensional, and hybrid models. Journal of Abnormal Psychology, 125(5), 715–726. 10.1037/abn0000174. 27214062 PMC5050027

[r32] Lykken, D. T., Bouchard, T. J., McGue, M., & Tellegen, A. (1990). The Minnesota twin family registry: Some initial findings. Acta Geneticae Medicae et Gemellologiae, 39(1), 35–70. 10.1017/s0001566000005572. 2392892

[r33] Munn, M. A., Stallings, M. C., Rhee, S. H., Sobik, L. E., Corley, R. P., Rhea, S. A., & Hewitt, J. K. (2010). Bivariate analysis of disordered eating characteristics in adolescence and young adulthood. International Journal of Eating Disorders, 43, 751–761. 20957703 10.1002/eat.20854 PMC2980580

[r34] Myer, C., & Waller, G. (2001). Social convergence of disturbed eating attitudes in young adult women. Journal of Nervous and Mental Disorders, 189(2), 114–119. 10.1097/00005053-200102000-00007. 11225684

[r35] Neale, M. C., Boker, S.M., Xie, G., & Maes, H.H. (1995). Mx: Statistical modeling. Department of Psychiatry.

[r36] O’Connor, S. M., Burt, S. A., McGue, M., Iacono, W., & Klump, K. L. (2019). Elucidating factors underlying parent–offspring similarity in eating pathology in pre- and early puberty: Exploring the possibility of passive gene–environment correlation. Journal of Abnormal Psychology, 128(7), 658–670. 10.1037/abn0000466. 31535887 PMC6776685

[r37] O’Connor, S. M., Burt, S. A., VanHuysse, J. L., & Klump, K. L. (2016). What drives the association between weight conscious peer groups and disordered eating? Disentangling genetic and environmental selection from pure socialization. Journal of Abnormal Psychology, 125(3), 356–368. 10.1037/abn0000132. 27043917 PMC4824549

[r38] O’Connor, S. M., Culbert, K. M., Mayhall, L. A., Burt, S. A., & Klump, K. L. (2020). Differences in genetic and environmental influences on body weight and shape concerns across pubertal development in females. Journal of Psychiatric Research, 121, 39–46. 10.1016/j.jpsychires.2019.11.001. 31759219 PMC7099836

[r39] O’Connor, S. M., Mikhail, M., Anaya, C., Haller, L. L., Burt, S. A., McGue, M., Iacono, W. G., & Klump, K. L. (2022). Exploring the possibility of parents’ broad internalizing phenotype acting through passive gene-environment correlations on daughters’ disordered eating. Development and Psychopathology, 34(5), 1744–1755. 10.1017/S0954579422000608. 35983803 PMC9938845

[r40] Paxton, S. J., Schutz, H. K., Wertheim, E. H., & Muir, S. L. (1999). Friendship clique and peer influences on body image concerns, dietary restraint, extreme weight-loss behaviors, and binge eating in adolescent girls. Journal of Abnormal Psychology, 108(2), 255–266. 10.1037//0021-843x.108.2.255. 10369035

[r41] Peeters, H., Van Gestel, S., Vlietinck, R., Derom, C., & Derom, R. (1998). Validation of a telephone zygosity questionnaire in twins of known zygosity. Behavior Genetics, 28(3), 159–163. 10.1023/a:1021416112215. 9670591

[r42] Petersen, A. C., Crockett, L., Richards, M., & Boxer, A. (1988). A self-report measure of pubertal status: Reliability, validity, and initial norms. Journal of Youth Adolescence, 17(2), 117–133. 10.1007/BF01537962. 24277579

[r43] Podsakoff, P. M., MacKenzie, S. B., Lee, J., & Podsakoff, N. (2003). Common method biases in behavioral research: A critical review of the literature and recommended remedies. Journal of Applied Psychology, 88(5), 879–903. 10.1037/0021-9010.88.5.879. 14516251

[r44] Prnjak, K., Hay, P., Mond, J., Bussey, K., Trompeter, N., Lonergan, A., & Mitchison, D. (2021). The distinct role of body image aspects in predicting eating disorder onset in adolescents after one year. Journal of Abnormal Psychology, 130(3), 236–247. 10.1037/abn0000537. 33705157

[r45] Raftery, A. E. (1995). Bayesian model selection in social research. Sociological Methodology, 25, 111–163. 10.2307/271063.

[r46] Rayner, K. E., Schniering, C. A., Rapee, R. M., Taylor, A., & Hutchinson, D. M. (2013). Adolescent girls’ friendship networks, body dissatisfaction, and disordered eating: Examining selection and socialization processes. Journal of Abnormal Psychology, 122(1), 93–104. 10.1037/a0029304. 22867115

[r47] Reichborn-Kjennerud, T., Bulik, C. M., Kendler, K. S., Røysamb, E., Maes, H., Tambs, K., & Harris, J. R. (2003). Gender differences in binge-eating: A population-based twin study. Acta Psychiatrica Scandinavica, 108(3), 196–202. 10.1034/j.1600-0447.2003.00106.x. 12890274

[r48] Rutherford, J., McGuffin, P., Katz, R. J., & Murray, R. M. (1993). Genetic influences on eating attitudes in a normal female twin population. Psychological Medicine, 23(2), 425–436. 10.1017/s003329170002852x. 8332659

[r49] Schutz, H. K., Paxton, S. J., & Wertheim, E. H. (2002). Investigation of body comparison among adolescent girls. Journal of Applied Social Psychology, 32(9), 1906–1937. 10.1111/j.1559-1816.2002.tb00264.x.

[r50] Sclove, L. S. (1987). Application of model-selection criteria to some problems in multivariate analysis. Psychometrika, 52, 333–343. 10.1007/BF02294360.

[r51] Shroff, H., & Thompson, J. K. (2006). Peer influences, body-image dissatisfaction, eating dysfunction and self-esteem in adolescent girls. Journal of Health Psychology, 11(4), 533–551. 10.1177/1359105306065015. 16769734

[r52] Slof-Op’t Landt, M. C. T., Bartels, M., van Furth, E. F., van Beijsterveldt, C. E. M., Meulenbelt, I., Slagboom, P. E., & Boomsma, D. I. (2008). Genetic influences on disordered eating are largely independent of body mass index. Acta Psychiatrica Scandinavica, 117, 348–359. 18081919 10.1111/j.1600-0447.2007.01132.x

[r53] Spiegelhalter, D. J., Best, N. G., Carlin, B. P., & Van Der Linde, A. (2002). Bayesian measures of model complexity and fit. Journal of the Royal Statistical Society: Series B (Statistical Methodology), 64, 583–639. 10.1111/1467-9868.00353.

[r54] Stice, E., & Shaw, H. E. (2002). Role of body dissatisfaction in the onset and maintenance of eating pathology: A synthesis of research findings. Journal of Psychosomatic Research, 53(5), 985–993. 10.1016/s0022-3999(02)00488-9. 12445588

[r57] Taylor, C. B., Sharpe, T., Shisslak, C., Bryson, S., Estes, L. S., Gray, N., McKnight, K. M., Crago, M., Kraemer, H. C., & Killen, J. D. (1998). Factors associated with weight concerns in adolescent girls. International Journal of Eating Disorders, 24, 31–42. 10.1002/(sici)1098-108x(199807)24:1<31::aid-eat3>3.0.co;2-1. 9589309

[r66] Trogdon, J. G., Nonnemaker, J., & Pais, J. (2008). Peer effects in adolescent overweight. Journal of health economics, 27(5), 1388–1399. 18565605 10.1016/j.jhealeco.2008.05.003

[r58] Vander Wal, J. S., & Thelen, M. H. (2000). Predictors of body image dissatisfaction in elementary-age school girls. Eating Behaviors, 1, 105–122. 10.1016/s1471-0153(00)00011-8. 15001054

[r59] Van Huysse, J. L., Burt, S. A., O’Connor, S. M., Thompson, J. K., & Klump, K. L. (2016). Socialization and selection effects in the association between weight conscious peer groups and thin-ideal internalization: A co-twin control study. Body Image, 17, 1–9. 10.1016/j.bodyim.2016.01.005. 26859605 PMC4877246

[r61] von Ranson, K. M., Klump, K. L., Iacono, W. G., & McGue, M. (2005). The Minnesota eating behavior survey: A brief measure of disordered eating attitudes and behaviors. Eating Behaviors, 6, 373–392. 10.1016/j.eatbeh.2004.12.002. 16257811

[r62] Wade, T. D., Bulik, C. M., Neale, M., & Kendler, K. S. (2000). Anorexia nervosa and major depression: Shared genetic and environmental risk factors. The American Journal of Psychiatry, 157(3), 469–471. 10.1176/appi.ajp.157.3.469. 10698830

[r63] Wade, T. D., Martin, N. G., Neale, M. C., Tiggemann, M., Treloar, S. A., Bucholz, K. K., Madden, P. A. F., & Heath, A. C. (1999). The structure of genetic and environmental risk factors for three measures of disordered eating. Psychological Medicine, 29, 925–934. 10473319 10.1017/s0033291799008740

[r64] Wade, T. D., Wilkinson, J., & Ben-Tovim, D. (2003). The genetic epidemiology of body attitudes, the attitudinal component of body image in women. Psychological Medicine, 33, 1395–1405. 14672248 10.1017/s0033291703008572

