# Supplementary material for: The contribution of genetic and environmental influences underlying disordered eating to exposure to weight-conscious peers
Source: Psychol Med. 2026 Jan 9;56:e9. doi: 10.1017/S0033291725102948 (PMC12885350; doi:10.1017/S0033291725102948)
Supplement: O’Connor et al. supplementary material [file S0033291725102948sup001.docx]

Supplemental Table 1. Comparison of Bivariate Cholesky Decomposition Models in Additional Disordered Eating Constructs

| Model | *χ^2^* (*df*) | *χ^2^* diff (*df*)^a^ | *p* | AIC | *BIC* | *SABIC* | *DIC* |  | *χ^2^* (*df*) | *χ^2^* diff (*df*)^a^ | *p* | AIC | *BIC* | *SABIC* | *DIC* |
| --- | --- | --- | --- | --- | --- | --- | --- | --- | --- | --- | --- | --- | --- | --- | --- |
| **Pre-Early Pubertal Sample (n=276 twin pairs)** | | | | | | | |  | **Mid-Late Pubertal Sample (n=146 twin pairs)** | | | | | | |
| Body Dissatisfaction | |  |  |  |  |  |  |  |  |  |  |  |  |  |  |
| ACE | 2452.29(1071) |  |  | 310.29 | -1783.58 | -85.60 | -799.40 |  | 1389.75(555) |  |  | 279.75 | -686.17 | 191.94 | -176.16 |
| AE | 2460.72(1074) | 8.43(3) | .04 | 312.72 | -1787.79 | -85.06 | -800.85 |  | **1389.80(558)** | **.05(3)** | **.99** | **273.80** | **-693.61** | **189.25** | **-180.84** |
| CE | **2452.86(1074)** | **.57(3)** | **.90** | **304.86** | **-1791.73** | **-88.99** | **-804.79** |  | 1400.25(558) | 10.5(3) | .01 | 284.25 | -688.38 | 194.47 | -175.62 |
| E | 2537.72(1077) | 85.43(6) | <.01 | 383.72 | -1757.73 | -50.23 | -768.03 |  | 1452.51(561) | 62.76(6) | <.01 | 330.51 | -669.72 | 217.89 | -154.19 |
| Weight Preoccupation | |  |  |  |  |  |  |  |  |  |  |  |  |  |  |
| ACE | 2413.04(1072) |  |  | 269.04 | -1806.02 | -106.45 | -820.91 |  | 1325.86(554) |  |  | 217.86 | -715.62 | 160.90 | -206.53 |
| AE | 2429.43(1075) | 16.39(3) | <.01 | 279.43 | -1806.25 | -101.93 | -818.39 |  | **1327.29(557)** | **1.43(3)** | **.70** | **213.29** | **-722.37** | **158.90** | **-210.53** |
| CE | **2415.29(1075)** | **2.25(3)** | **.52** | **265.29** | **-1813.32** | **-109.00** | **-825.46** |  | 1333.82(557) | 7.96(3) | .05 | 219.82 | -719.11 | 162.16 | -207.26 |
| E | 2515.50(1078) | 102.46(6) | <.01 | 359.50 | -1771.65 | -62.57 | -781.03 |  | 1389.91(560) | 64.05(6) | <.01 | 269.91 | -698.53 | 187.49 | -183.93 |
| Binge Eating | |  |  |  |  |  |  |  |  |  |  |  |  |  |  |
| ACE | 2445.23(1075) |  |  | 295.23 | -1798.35 | -94.03 | -810.49 |  | 1373.80(556) |  |  | 261.80 | -696.63 | 183.06 | -185.70 |
| AE | 2453.66(1078) | 8.43(3) | .04 | 297.66 | -1802.57 | -93.49 | -811.95 |  | **1373.89(559)** | **.09(3)** | **.99** | **255.89** | **-704.05** | **180.39** | **-190.37** |
| CE | **2447.41(1078)** | **2.18(3)** | **.54** | **291.41** | **-1805.69** | **-96.61** | **-815.07** |  | 1387.63(559) | 13.83(3) | <.01 | 269.63 | -697.18 | 187.26 | -183.50 |
| E | 2556.45(1081) | 111.22(6) | <.01 | 394.45 | -1759.60 | -45.77 | -766.23 |  | 1427.55(562) | 53.75(6) | <.01 | 303.55 | -684.69 | 204.50 | -168.25 |
| Eating in the Absence of Hunger | | |  |  |  |  |  |  |  |  |  |  |  |  |  |
| ACE | 2473.99(1069) |  |  | 335.99 | -1767.11 | -72.30 | -784.76 |  | 1369.37(554) |  |  | 261.37 | -693.87 | 182.65 | -184.78 |
| AE | 2484.70(1072) | 10.71(3) | .01 | 340.70 | -1770.19 | -70.62 | -785.09 |  | **1369.48(557)** | **.11(3)** | **.99** | **255.48** | **-701.28** | **179.99** | **-189.43** |
| CE | **2474.20(1072)** | **.21(3)** | **.98** | **330.20** | **-1775.44** | **-75.87** | **-790.33** |  | 1378.53(557) | 9.16(3) | .03 | 264.53 | -696.75 | 184.52 | -184.91 |
| E | 2559.02(1075) | 85.03(6) | <.01 | 409.02 | -1741.46 | -37.13 | -753.60 |  | 1427.74(560) | 58.37(6) | <.01 | 307.74 | -679.62 | 206.40 | -165.01 |
| Emotional Eating | |  |  |  |  |  |  |  |  |  |  |  |  |  |  |
| ACE | 2402.68(1031) |  |  | 340.68 | -1695.98 | -61.41 | -748.55 |  | 1351.46(541) |  |  | 269.46 | -670.48 | 185.48 | -173.33 |
| AE | 2410.63(1034) | 7.95(3) | .05 | 342.63 | -1700.43 | -61.11 | -750.25 |  | **1352.12(544)** | **.66(3)** | **.88** | **264.12** | **-677.61** | **183.09** | **-177.71** |
| CE | **2403.56(1034)** | .88(3) | **.83** | **335.56** | **-1703.97** | **-64.65** | **-753.79** |  | 1359.34(544) | 7.88(3) | .05 | 271.34 | -674.00 | 186.70 | -174.10 |
| E | 2472.22(1037) | 69.54(6) | <.01 | 398.22 | -1678.07 | -33.99 | -725.13 |  | 1394.52(547) | 43.06(6) | <.01 | 300.52 | -663.88 | 201.58 | -161.22 |

*Note.* AIC= Akaike’s information criterion; BIC= Bayesian information criterion; SABIC= Sample-size adjusted Bayesian information criterion; DIC= Deviance information criterion; *χ^2^* diff = chi-squared difference test. The best fitting model is indicated in boldface.

^a^AE, CE, and E models are compared with the full ACE model for the *χ^2^* diff test.

Supplemental Table 2. Parameter Estimates for Bivariate Cholesky Model for Body Dissatisfaction and Exposure to Weight-Conscious Peers Composite Score (Pre-early puberty: 275 twin pairs; Mid-late puberty: 144 twin pairs)

|  | Pre-Pubertal Sample | | Pubertal Sample | |
| --- | --- | --- | --- | --- |
|  | Full Model:  ACE | Best Fitting: CE | Full Model:  ACE | Best Fitting: AE |
| **Body Dissatisfaction** | | | | |
| **Bivariate correlations** |  |  |  |  |
| Additive Genetic (r_a_) | .57 (-1.00, 1.00) | - | .45 (-1.00, 1.00) | **.47 (.24, .67)** |
| Shared Environmental (r_c_) | .27 (-1.00, 1.00) | **.33 (.07, .55)** | 1.00 (-1.00, 1.00) | - |
| Non-shared Environmental (r_e_) | **.25 (.10, .39)** | **.26 (.15, .37)** | **.37 (.15, .55)** | **.36 (.16, .54)** |
|  |  |  |  |  |
| **Cholesky decomposition models** | |  |  |  |
| Heritability estimate |  |  |  |  |
| Body Dissatisfaction |  |  |  |  |
| Total (h^2^) | .14 (.00, .43) | - | .44 (.00, .63) | **.50 (.33, .63)** |
| Peer Composite Score |  |  |  |  |
| Total (h^2^) | .08 (.00, .42) | - | **.63 (.21, .76)** | **.65 (.50, .76)** |
| Attributable (h^2^_a_) | .02 (.00, .41) | - | .13 (.00, .72) | **.15 (.04, .31)** |
| Residual (h^2^_r_) | .05 (.00, .38) | - | .50 (.00, .63) | **.50 (.34, .64)** |
| % Attributable | 25.0% | - | 20.6% | 23.1% |
|  |  |  |  |  |
| Shared environmentality estimate | |  |  |  |
| Body Dissatisfaction |  |  |  |  |
| Total (c^2^) | .16 (.00, .36) | **.26 (.15, .37)** | .05 (.00, .47) | - |
| Peer Composite Score |  |  |  |  |
| Total (c^2^) | **.41 (.12, .55)** | **.47 (.37, .56)** | .02 (.00, .37) | - |
| Attributable (c^2^_a_) | .03 (.00, .54) | .05 (.00, .15) | .02 (.00, .37) | - |
| Residual (c^2^_r_) | .38 (.00, .51) | **.42 (.31, .51)** | .00 (.00, .28) | - |
| % Attributable | 7.3% | 10.6% | 100% | - |
|  |  |  |  |  |
| Non-shared environmentality estimate | |  |  |  |
| Body Dissatisfaction |  |  |  |  |
| Total (e^2^) | **.70 (.56, .84)** | **.74 (.63, .85)** | **.51 (.37, .69)** | **.50 (.37, .67)** |
| Peer Composite Score |  |  |  |  |
| Total (e^2^) | **.51 (.40, .63)** | **.53 (.44, .63)** | **.35 (.24, .51)** | **.35 (.24, .50)** |
| Attributable (e^2^_a_) | **.03 (.01, .08)** | **.04 (.01, .08)** | **.05 (.01, .13)** | **.05 (.01, .12)** |
| Residual (e^2^_r_) | **.48 (.37, .59)** | **.50 (.41, .59)** | **.30 (.21, .45)** | **.30 (.21, .44)** |
| % Attributable | 5.9% | 7.5% | 14.3% | 14.3% |

*Note.* h^2^= total heritability; h^2^_a_= heritability of the peer composite score that is attributable to genetic effects of body dissatisfaction; h^2^_r_= heritability of peer composite score that is independent of genetic effects of body dissatisfaction; c^2^= shared environmentality; c^2^_a_= shared environmentality of the peer composite score that is attributable to common shared environmental effects of body dissatisfaction; c^2^_r_= shared environmentality of the peer composite score that is independent of shared environmental effects of body dissatisfaction; e^2^= non-shared environmentality; e^2^_a_= non-shared environmentality of the peer composite score that is attributable to common non-shared environmental effects of body dissatisfaction; e^2^_r_= non-shared environmentality of the peer composite score that is independent of non-shared environmental effects of body dissatisfaction. Body dissatisfaction was assessed via the body dissatisfaction subscale of the Minnesota Eating Behavior Survey (von Ranson, Klump, Iacono & McGue, 2005).

Supplemental Table 3. Parameter Estimates for Bivariate Cholesky Model for Weight Preoccupation and Exposure to Weight-Conscious Peers Composite Score (Pre-early puberty: 275 twin pairs; Mid-late puberty: 144 twin pairs)

|  | Pre-Pubertal Sample | | Pubertal Sample | |
| --- | --- | --- | --- | --- |
|  | Full Model: ACE | Best Fitting: CE | Full Model: ACE | Best Fitting: AE |
| **Weight Preoccupation** | | | | |
| **Bivariate correlations** |  |  |  |  |
| Additive Genetic (r_a_) | -1.00 (-1.00, 1.00) | - | .79 (-1.00, 1.00) | **.57 (.36, .74)** |
| Shared Environmental (r_c_) | **.85 (.48, 1.00)** | **.61 (.44, .75)** | .25 (-1.00, 1.00) | - |
| Non-shared Environmental (r_e_) | **.36 (.23, .48)** | **.31 (.20, .42)** | **.36 (.15, .55)** | **.37 (.17, .54)** |
|  |  |  |  |  |
| **Cholesky decomposition models** |  |  |  |  |
| Heritability estimate |  |  |  |  |
| Weight Preoccupation |  |  |  |  |
| Total (h^2^) | .03 (.00, .23) | - | .23 (.00, .60) | **.48 (.31, .62)** |
| Peer Composite Score |  |  |  |  |
| Total (h^2^) | .16 (.00, .44) | - | **.63 (.19, .76)** | **.66 (.51, .76)** |
| Attributable (h^2^_a_) | .16 (.00, .44) | - | .39 (.00, .75) | **.21 (.08, .38)** |
| Residual (h^2^_r_) | .00 (.00, .43) | - | .23 (.00, .57) | **.45 (.29, .58)** |
| % Attributable | 100% | - | 61.9% | 31.8% |
|  |  |  |  |  |
| Shared environmentality estimate |  |  |  |  |
| Weight Preoccupation |  |  |  |  |
| Total (c^2^) | **.38 (.19, .49)** | **.41 (.30, .50)** | .23 (.00, .49) | - |
| Peer Composite Score |  |  |  |  |
| Total (c^2^) | **.35 (.11, .52)** | **.46 (.37, .55)** | .03 (.00, .39) | - |
| Attributable (c^2^_a_) | **.25 (.07, .49)** | **.17 (.08, .30)** | .00 (.00, .32) | - |
| Residual (c^2^_r_) | .10 (.00, .36) | **.29 (.19, .39)** | .03 (.00, .33) | - |
| % Attributable | 71.4% | 37.0% | 0% | - |
|  |  |  |  |  |
| Non-shared environmentality estimate | |  |  |  |
| Weight Preoccupation |  |  |  |  |
| Total (e^2^) | **.59 (.49, .69)** | **.59 (.50, .70)** | **.54 (.39, .72)** | **.52 (.38, .69)** |
| Peer Composite Score |  |  |  |  |
| Total (e^2^) | **.49 (.39, .61)** | **.54 (.45, .63)** | **.35 (.24, .50)** | **.34 (.24, .49)** |
| Attributable (e^2^_a_) | **.06 (.03, .12)** | **.05 (.02, .10)** | **.05 (.01, .12)** | **.05 (.01, .12)** |
| Residual (e^2^_r_) | **.43 (.33, .54)** | **.48 (.40, .58)** | **.30 (.21, .44)** | **.29 (.21, .42)** |
| % Attributable | 12.2% | 9.3% | 14.3% | 14.7% |

*Note.* h^2^= total heritability; h^2^_a_= heritability of the peer composite score that is attributable to genetic effects of weight preoccupation; h^2^_r_= heritability of peer composite score that is independent of genetic effects of weight preoccupation; c^2^= shared environmentality; c^2^_a_= shared environmentality of the peer composite score that is attributable to common shared environmental effects of weight preoccupation; c^2^_r_= shared environmentality of the peer composite score that is independent of shared environmental effects of weight preoccupation; e^2^= non-shared environmentality; e^2^_a_= non-shared environmentality of the peer composite score that is attributable to common non-shared environmental effects of weight preoccupation; e^2^_r_= non-shared environmentality of the peer composite score that is independent of non-shared environmental effects of weight preoccupation. Weight preoccupation was assessed via the weight preoccupation subscale of the Minnesota Eating Behavior Survey (von Ranson, Klump, Iacono & McGue, 2005).

Supplemental Table 4. Parameter Estimates for Bivariate Cholesky Model for Binge Eating and Exposure to Weight-Conscious Peers Composite Score (Pre-early puberty: 275 twin pairs; Mid-late puberty: 144 twin pairs)

|  | Pre-Pubertal Sample | | Pubertal Sample | |
| --- | --- | --- | --- | --- |
|  | Full Model: ACE | Best Fitting: CE | Full Model: ACE | Best Fitting: AE |
| **Binge Eating** | | | | |
| **Bivariate correlations** |  |  |  |  |
| Additive Genetic (r_a_) | .34 (-1.00, 1.00) | - | **.63 (.26, 1.00)** | **.59 (.34, .82)** |
| Shared Environmental (r_c_) | .43 (-1.00, 1.00) | **.38 (.18, .55)** | -1.00 (-1.00, 1.00) | - |
| Non-shared Environmental (r_e_) | **.39 (.24, .53)** | **.38 (.27, .48)** | .13 (-.10, .35) | .14 (-.08, .35) |
|  |  |  |  |  |
| **Cholesky decomposition models** |  |  |  |  |
| Heritability estimate |  |  |  |  |
| Binge Eating |  |  |  |  |
| Total (h^2^) | .26 (.00, .55) | - | **.44 (.15, .60)** | **.45 (.25, .60)** |
| Peer Composite Score |  |  |  |  |
| Total (h^2^) | .08 (.00, .43) | - | **.59 (.17, .76)** | **.65 (.50, .76)** |
| Attributable (h^2^_a_) | .01 (.00, .33) | - | **.24 (.03, .69)** | **.22 (.07, .44)** |
| Residual (h^2^_r_) | .07 (.00, .37) | - | .36 (.00, .60) | **.43 (.21, .59)** |
| % Attributable | 12.5% | - | 40.7% | 33.8% |
|  |  |  |  |  |
| Shared environmentality estimate |  |  |  |  |
| Binge Eating |  |  |  |  |
| Total (c^2^) | .19 (.00, .46) | **.39 (.28, .48)** | .00 (.00, .20) | - |
| Peer Composite Score |  |  |  |  |
| Total (c^2^) | **.40 (.11, .55)** | **.46 (.36, .55)** | .05 (.00, .39) | - |
| Attributable (c^2^_a_) | .08 (.00, .53) | **.07 (.01, .15)** | .05 (.00, .39) | - |
| Residual (c^2^_r_) | .33 (.00, .48) | **.40 (.30, .48)** | .00 (.00, .38) | - |
| % Attributable | 20.0% | 15.2% | 100% | - |
|  |  |  |  |  |
| Non-shared environmentality estimate | |  |  |  |
| Binge Eating |  |  |  |  |
| Total (e^2^) | **.55 (.44, .69)** | **.61 (.52, .72)** | **.55 (.40, .75)** | **.55 (.40, .75)** |
| Peer Composite Score |  |  |  |  |
| Total (e^2^) | **.52 (.40, .63)** | **.54 (.45, .64)** | **.35 (.25, .52)** | **.35 (.24, .50)** |
| Attributable (e^2^_a_) | **.08 (.03,.16)** | **.08 (.04, .13)** | .01 (.00, .05) | .01 (.00, .05) |
| Residual (e^2^_r_) | **.44 (.34, .54)** | **.46 (.38, .55)** | **.35 (.24, .51)** | **.34 (.24, .49)** |
| % Attributable | 15.4% | 14.8% | 2.9% | 2.9% |
|  |  |  |  |  |

*Note.* h^2^= total heritability; h^2^_a_= heritability of the peer composite score that is attributable to genetic effects of binge eating; h^2^_r_= heritability of peer composite score that is independent of genetic effects of binge eating; c^2^= shared environmentality; c^2^_a_= shared environmentality of the peer composite score that is attributable to common shared environmental effects of binge eating; c^2^_r_= shared environmentality of the peer composite score that is independent of shared environmental effects of binge eating; e^2^= non-shared environmentality; e^2^_a_= non-shared environmentality of the peer composite score that is attributable to common non-shared environmental effects of binge eating; e^2^_r_= non-shared environmentality of the peer composite score that is independent of non-shared environmental effects of binge eating. Binge eating was assessed via the binge eating subscale of the Minnesota Eating Behavior Survey (von Ranson, Klump, Iacono & McGue, 2005).

Supplemental Table 5. Parameter Estimates for Bivariate Cholesky Model for Eating in the Absence of Hunger and Exposure to Weight-Conscious Peers Composite Score (Pre-early puberty: 275 twin pairs; Mid-late puberty: 144 twin pairs)

|  | Pre-Pubertal Sample | | Pubertal Sample | |
| --- | --- | --- | --- | --- |
|  | Full Model: ACE | Best Fitting: CE | Full Model: ACE | Best Fitting: AE |
| **Eating in the Absence of Hunger** | | | | |
| **Bivariate correlations** |  |  |  |  |
| Additive Genetic (r_a_) | 1.00 (-1.00, 1.00) | - | .48 (-1.00, 1.00) | **.42 (.17, .65)** |
| Shared Environmental (r_c_) | .43 (-1.00, 1.00) | **.49 (.28, .69)** | -.15 (-1.00, 1.00) | - |
| Non-shared Environmental (r_e_) | **.23 (.10, .36)** | **.24 (.13, .35)** | .22 (-.01, .43) | **.23 (.01, .42)** |
|  |  |  |  |  |
| **Cholesky decomposition models** |  |  |  |  |
| Heritability estimate |  |  |  |  |
| Eating in the Absence of Hunger |  |  |  |  |
| Total (h^2^) | .04 (.00, .40) | - | .39 (.00, .61) | **.46 (.28, .61)** |
| Peer Composite Score |  |  |  |  |
| Total (h^2^) | .07 (.00, .41) | - | **.64 (.19, .76)** | **.65 (.50, .76)** |
| Attributable (h^2^_a_) | .07 (.00, .41) | - | .14 (.00, .74) | **.12 (.02, .28)** |
| Residual (h^2^_r_) | .00 (.00, .32) | - | .49 (.00, .67) | **.53 (.36, .67)** |
| % Attributable | 100% | - | 21.9% | 18.5% |
|  |  |  |  |  |
| Shared environmentality estimate |  |  |  |  |
| Eating in the Absence of Hunger |  |  |  |  |
| Total (c^2^) | .27 (.00, .40) | .**30 (.19, .41)** | .06 (.00, .43) | - |
| Peer Composite Score |  |  |  |  |
| Total (c^2^) | **.42 (.13, .55)** | **.47 (.37, .56)** | .01 (.00, .38) | - |
| Attributable (c^2^_a_) | .08 (.00, .50) | **.11 (.03. .23)** | .00 (.00, .37) | - |
| Residual (c^2^_r_) | .34 (.00, .46) | **.35 (.24, .46)** | .01 (.00, .35) | - |
| % Attributable | 19.0% | 23.4% | 0% | - |
|  |  |  |  |  |
| Non-shared environmentality estimate | |  |  |  |
| Eating in the Absence of Hunger |  |  |  |  |
| Total (e^2^) | **.69 (.57, .81)** | **.70 (.60, .81)** | **.55 (.39, .75)** | **.54 (.39, .72)** |
| Peer Composite Score |  |  |  |  |
| Total (e^2^) | **.52 (.40, .63)** | **.53 (.44, .63)** | **.35 (.24, .51)** | **.35 (.24, .50)** |
| Attributable (e^2^_a_) | .03 (.00, .07) | **.03 (.01, .07)** | .02 (.00, .08) | .02 (.00, .07) |
| Residual (e^2^_r_) | **.49 (.38, .59)** | **.50 (.42, .60)** | **.33 (.23, .49)** | **.33 (.23, .48)** |
| % Attributable | 5.8% | 5.7% | 5.7% | 5.7% |
|  |  |  |  |  |

*Note.* h^2^= total heritability; h^2^_a_= heritability of the peer composite score that is attributable to genetic effects of eating in the absence of hunger; h^2^_r_= heritability of peer composite score that is independent of genetic effects of eating in the absence of hunger; c^2^= shared environmentality; c^2^_a_= shared environmentality of the peer composite score that is attributable to common shared environmental effects of eating in the absence of hunger; c^2^_r_= shared environmentality of the peer composite score that is independent of shared environmental effects of eating in the absence of hunger; e^2^= non-shared environmentality; e^2^_a_= non-shared environmentality of the peer composite score that is attributable to common non-shared environmental effects of eating in the absence of hunger; e^2^_r_= non-shared environmentality of the peer composite score that is independent of non-shared environmental effects of eating in the absence of hunger. Eating in the absence of hunger was assessed using the 14-item self-report questionnaire, Eating in the Absence of Hunger for Children and Adolescence (EAH-Child). EAH-Child was developed to assess precipitants to eating when not hungry in children and adolescents age 6 to 19 (Tanofsky-Kraff et al., 2008).

Supplemental Table 6. Parameter Estimates for Bivariate Cholesky Model for Emotional Eating and Exposure to Weight-Conscious Peers Composite Score (Pre-early puberty: 275 twin pairs; Mid-late puberty: 144 twin pairs)

|  | Pre-Pubertal Sample | | Pubertal Sample | |
| --- | --- | --- | --- | --- |
|  | Full Model: ACE | Best Fitting: CE | Full Model: ACE | Best Fitting: AE |
| **Emotional Eating** | | | | |
| **Bivariate correlations** |  |  |  |  |
| Additive Genetic (r_a_) | 1.00 (-1.00, 1.00) | - | 1.00 (-1.00, 1.00) | **.42 (.17, .65)** |
| Shared Environmental (r_c_) | 1.00 (-.53, 1.00) | **.57 (.25, 1.00)** | .98 (-1.00, 1.00) | **-** |
| Non-shared Environmental (r_e_) | **.25 (.12, .39)** | **.28 (.16, .39)** | .22 (-.01, .42) | **.23 (.01, .42)** |
|  |  |  |  |  |
| **Cholesky decomposition models** |  |  |  |  |
| Heritability estimate |  |  |  |  |
| Emotional Eating |  |  |  |  |
| Total (h^2^) | .17 (.00, .32) | - | .02 (.00, .31) | .13 (.00. .33) |
| Peer Composite Score |  |  |  |  |
| Total (h^2^) | .09 (.00, .42) | - | **.64 (.20, .76)** | **.65 (.51, .76)** |
| Attributable (h^2^_a_) | .09 (.00, .42) | - | .64 (.00, .76) | .17 (.00, .75) |
| Residual (h^2^_r_) | .00 (.00, .32) | - | .00 (.00, .70) | .49 (.00, .70) |
| % Attributable | 100% | - | 100% | 26.2% |
|  |  |  |  |  |
| Shared environmentality estimate |  |  |  |  |
| Emotional Eating |  |  |  |  |
| Total (c^2^) | .01 (.00, .24) | .14 (.02, .26) | .11 (.00, .28) | - |
| Peer Composite Score |  |  |  |  |
| Total (c^2^) | **.40 (.12, .55)** | **.47 (.37, .56)** | .01 (.00, .38) | - |
| Attributable (c^2^_a_) | .40 (.00, .55) | .**15 (.03, .52)** | .01 (.00, .35) | - |
| Residual (c^2^_r_) | .00 (.00, .45) | .31 (.00, .45) | .00 (.00. .35) | - |
| % Attributable | 100% | 31.9% | 100% | - |
|  |  |  |  |  |
| Non-shared environmentality estimate | |  |  |  |
| Emotional Eating |  |  |  |  |
| Total (e^2^) | **.82 (.67, .98)** | **.86 (.74, .98)** | **.87 (.69, 1.00)** | **.87 (.67, 1.00)** |
| Peer Composite Score |  |  |  |  |
| Total (e^2^) | **.51 (.40, .62)** | **.53 (.44, .63)** | **.35 (.24, .51)** | **.35 (.24, .50)** |
| Attributable (e^2^_a_) | **.03 (.01, .08)** | **.04 (.01, .09)** | .02 (.00, .07) | .02 (.00, .06) |
| Residual (e^2^_r_) | **.48 (.37, .58)** | **.49 (.41, .58)** | **.33 (.23, .49)** | **.33 (.23, .48)** |
| % Attributable | 5.9% | 7.5% | 5.7% | 5.7% |

*Note.* h^2^= total heritability; h^2^_a_= heritability of the peer composite score that is attributable to genetic effects of emotional eating; h^2^_r_= heritability of peer composite score that is independent of genetic effects of emotional eating; c^2^= shared environmentality; c^2^_a_= shared environmentality of the peer composite score that is attributable to common shared environmental effects of emotional eating; c^2^_r_= shared environmentality of the peer composite score that is independent of shared environmental effects of emotional eating; e^2^= non-shared environmentality; e^2^_a_= non-shared environmentality of the peer composite score that is attributable to common non-shared environmental effects of emotional eating; e^2^_r_= non-shared environmentality of the peer composite score that is independent of non-shared environmental effects of emotional eating. Emotional eating was assessed via the 26-item, self-report questionnaire, Emotional Eating Scale-Adapted for Children and Adolescents (EES-C). The EES-C assesses the urge to cope with negative affect through eating (Tanofsky-Kraff et al., 2007).

Supplemental Table 7. Comparison of Bivariate Cholesky Decomposition Models in Separately in each Peer Exposure Questionnaire

| Model | *χ^2^* (*df*) | *χ^2^* diff (*df*)^a^ | *p* | AIC | *BIC* | *SABIC* | *DIC* |  | *χ^2^* (*df*) | *χ^2^* diff (*df*)^a^ | *p* | AIC | *BIC* | *SABIC* | *DIC* |
| --- | --- | --- | --- | --- | --- | --- | --- | --- | --- | --- | --- | --- | --- | --- | --- |
| **Pre-Early Pubertal Sample (n=275 twin pairs)** | | | | | | | |  | **Mid-Late Pubertal Sample (n=144 twin pairs)** | | | | | | |
| Perceived Friend Preoccupation with Weight and Dieting Scale | | | | | |  |  |  |  |  |  |  |  |  |  |
| ACE | 2600.69(1042) |  |  | 516.69 | -1626.00 | 26.00 | -668.46 |  | 1490.92(531) |  |  | 428.92 | -572.18 | 267.91 | -84.22 |
| AE | 2609.79(1045) | 9.10(3) | .03 | 519.79 | -1629.87 | 26.88 | -669.58 |  | **1492.52(534)** | **1.60(3)** | **.66** | **424.52** | **-578.82** | **266.01** | **-88.11** |
| CE | **2602.12(1045)** | **1.43(3)** | **.70** | **512.12** | **-1633.70** | **23.05** | **-673.41** |  | 1497.66(534) | 6.74(3) | .08 | 429.66 | -576.25 | 268.58 | -85.54 |
| E | 2679.67(1048) | 78.98(6) | <.01 | 583.67 | -1603.35 | 58.15 | -640.31 |  | 1555.75(537) | 64.83(6) | <.01 | 481.75 | -554.65 | 294.93 | -61.18 |
| Appearance Conversations with Friends | | | | | | |  |  |  |  |  |  |  |  |  |
| ACE | 2612.22(1066) |  |  | 480.22 | -1687.63 | 2.41 | -708.04 |  | 1551.55(552) |  |  | 447.55 | -593.97 | 279.34 | -86.72 |
| AE | 2618.56(1069) | 6.34(3) | .10 | 480.56 | -1692.89 | 1.91 | -710.54 |  | **1551.81(555)** | **0.26(3)** | **.97** | **441.81** | **-601.29** | **276.77** | **-91.28** |
| CE | **2615.74(1069)** | **3.52(3)** | **.32** | **477.74** | **-1694.29** | **0.50** | **-711.95** |  | 1564.50(555) | 12.95(3) | <.01 | 454.50 | -594.94 | 283.11 | -84.93 |
| E | 2709.90(1072) | 97.68(6) | <.01 | 565.90 | -1655.64 | 43.91 | -670.54 |  | 1627.54(558) | 75.99(6) | <.01 | 511.54 | -570.86 | 311.94 | -58.09 |
| Friends as a Source of Influence Scale | | | | | | | |  |  |  |  |  |  |  |  |
| ACE | 2716.99(1059) |  |  | 598.99 | -1615.59 | 63.36 | -642.43 |  | 1527.39(548) |  |  | 431.39 | -596.12 | 270.86 | -92.55 |
| AE | **2721.25(1062)** | **4.26(3)** | **.23** | **597.25** | **-1621.88** | **61.82** | **-645.97** |  | **1528.43(551)** | **1.04 (3)** | **.79** | **426.43** | **-603.05** | **268.68** | **-96.71** |
| CE | **2727.15(1062)** | **10.16(3)** | **.02** | **603.15** | **-1618.93** | **64.77** | **-643.02** |  | 1533.41(551) | 6.02(3) | .11 | 431.41 | -600.56 | 271.17 | -94.23 |
| E | 2795.55(1065) | 78.56(6) | <.01 | 665.55 | -1593.16 | 95.30 | -614.49 |  | 1566.56(554) | 39.17(6) | <.01 | 458.56 | -591.43 | 285.04 | -82.34 |
| Peer Attribution Scale | |  |  |  |  |  |  |  |  |  |  |  |  |  |  |
| ACE | 2691.36(1064) |  |  | 563.36 | -1642.44 | 44.43 | -664.69 |  | 1543.91(549) |  |  | 445.91 | -590.35 | 278.22 | -85.85 |
| AE | 2698.81(1067) | 7.45(3) | .06 | 564.81 | -1647.14 | 44.48 | -666.64 |  | **1543.91(552)** | **0.00(3)** | **1.00** | **439.91** | **-597.79** | **275.52** | **-90.54** |
| CE | **2695.25(1067)** | 3.89(3) | **.27** | **561.25** | **-1648.92** | **42.71** | **-668.41** |  | 1556.68(552) | 12.77(3) | .01 | 452.68 | -591.40 | 281.91 | -84.15 |
| E | 2789.66(1070) | 98.30(6) | <.01 | 649.66 | -1610.14 | 86.24 | -626.88 |  | 1594.29(555) | 50.38(6) | <.01 | 484.29 | -580.04 | 298.01 | -70.03 |

*Note.* AIC= Akaike’s information criterion; BIC= Bayesian information criterion; SABIC= Sample-size adjusted Bayesian information criterion; DIC= Deviance information criterion; *χ^2^* diff = chi-squared difference test. The best fitting model is indicated in boldface.

^a^AE, CE, and E models are compared with the full ACE model for the *χ^2^* diff test.

Supplemental Table 8. Parameter Estimates for Bivariate Model for Disordered Eating and the Perceived Friend Preoccupation with Weight and Dieting Scale

|  | Pre-Pubertal Sample | | Pubertal Sample | |
| --- | --- | --- | --- | --- |
|  | Full Model: ACE | Best Fitting: CE | Full Model: ACE | Best Fitting: AE |
| **Bivariate correlations** |  |  |  |  |
| Additive Genetic (r_a_) | -1.00 (-1.00, 1.00) | - | .50 (-1.00, 1.00) | **.36 (.09, .56)** |
| Shared Environmental (r_c_) | **.85 (.36, 1.00)** | **.62 (.43, .78)** | -1.00 (-1.00, 1.00) | - |
| Non-shared Environmental (r_e_) | **.39 (.25, .51)** | **.34 (.23, .45)** | .**41 (.20, .59)** | **.42 (.22, .58)** |
|  |  |  |  |  |
| **Cholesky decomposition models** | |  |  |  |
| Heritability estimate |  |  |  |  |
| MEBS Total Score |  |  |  |  |
| Total (h^2^) | .10 (.00, .42) | - | **.52 (.10, .65)** | **.52 (.36, .65)** |
| PFP |  |  |  |  |
| Total (h^2^) | .05 (.00, .38) | - | .31 (.00, .68) | **.58 (.41, .70)** |
| Attributable (h^2^_a_) | .05 (.00, .36) | - | .08 (.00, .08) | .07 (.00, .20) |
| Residual (h^2^_r_) | <.01 (.00, .36) | - | .23 (.00, .60) | **.51 (.36, .63)** |
| % Attributable | 100% | - | 25.8% | 12.1% |
|  |  |  |  |  |
| Shared environmentality estimate | |  |  |  |
| MEBS Total Score |  |  |  |  |
| Total (c^2^) | **.33 (.06, .48)** | **.40 (.30, .50)** | <.00 (.00, .35) | - |
| PFP |  |  |  |  |
| Total (c^2^) | **.36 (.08, .49)** | **.39 (.28, .49)** | .25 (.00, .57) | - |
| Attributable (c^2^_a_) | **.26 (.03, .47)** | **.15 (.07, .26)** | .25 (.00, .77) | - |
| Residual (c^2^_r_) | .10 (.00, .32) | **.24 (.14, .34)** | <.01 (.00, .53) | - |
| % Attributable | 72.2% | 38.4% | 100% | - |
|  |  |  |  |  |
| Non-shared environmentality estimate | |  |  |  |
| MEBS Total Score |  |  |  |  |
| Total (e^2^) | **.57 (.45, .69)** | **.60 (.50, .70)** | **.48 (.35, .64)** | **.48 (.35, .64)** |
| PFP |  |  |  |  |
| Total (e^2^) | **.59(.47, .71)** | **.61 (.51, .72)** | **.44 (.30, .64)** | **.42 (.30, .59)** |
| Attributable (e^2^_a_) | **.09 (.04, .16)** | **.07 (.03, .13)** | **.07 (.02, .19)** | **.07 (.02, .17)** |
| Residual (e^2^_r_) | **.50 (.39, .62)** | **.54 (.45, .64)** | **.37 (.25, .53)** | **.35 (.24, .49)** |
| % Attributable | 15.2% | 11.5% | 15.9% | 17.1% |

*Note.* MEBS= Minnesota Eating Behaviors Survey; PFP= Perceived Friend Preoccupation with Weight and Dieting Scale; h^2^= total heritability; h^2^_a_= heritability of PFP that is attributable to genetic effects of MEBS Total Score; h^2^_r_= heritability of PFP that is independent of genetic effects of MEBS Total Score; c^2^= shared environmentality; c^2^_a_= shared environmentality of PFP that is attributable to common shared environmental effects of MEBS Total Score; c^2^_r_= shared environmentality of PFP that is independent of shared environmental effects of MEBS Total Score; e^2^= non-shared environmentality; e^2^_a_= non-shared environmentality of PFP that is attributable to common non-shared environmental effects of MEBS Total Score; e^2^_r_= non-shared environmentality of PFP that is independent of non-shared environmental effects of MEBS Total Score; % Attributable = the percentage of variance attributable to MEBS Total Score out of the total variance underlying PFP

Supplemental Table 9. Parameter Estimates for Bivariate Model for Disordered Eating and the Appearance Conversations with Friends Questionnaire

|  | Pre-Pubertal Sample | | Pubertal Sample | |
| --- | --- | --- | --- | --- |
|  | Full Model: ACE | Best Fitting: CE | Full Model: ACE | Best Fitting: AE |
| **Bivariate correlations** |  |  |  |  |
| Additive Genetic (r_a_) | -1.00 (-1.00, 1.00) | - | **.47 (.05, 1.00)** | **.41 (.18, .60)** |
| Shared Environmental (r_c_) | **.71 (.16, 1.00)** | **.32 (.10, .51)** | -1.00 (-1.00, 1.00) | - |
| Non-shared Environmental (r_e_) | **.41 (.26, .52)** | **.34 (.23, .44)** | .22 (.00, .42) | **.22 (.01, .42)** |
|  |  |  |  |  |
| **Cholesky decomposition models** | |  |  |  |
| Heritability estimate |  |  |  |  |
| MEBS Total Score |  |  |  |  |
| Total (h^2^) | .09 (.00, .46) | - | **.53 (.13, .66)** | **.53 (.37, .66)** |
| ACF |  |  |  |  |
| Total (h^2^) | .15 (.00, .50) | - | **.59 (.18, .77)** | **.68 (.54, .77)** |
| Attributable (h^2^_a_) | .15 (.00, .48) | - | .13 (.00, .65) | **.11 (.02, .25)** |
| Residual (h^2^_r_) | <.01 (.00, .49) | - | .46 (.00, .68) | **.56 (.41, .69)** |
| % Attributable | 100% | - | 22.0% | 16.2 % |
|  |  |  |  |  |
| Shared environmentality estimate | |  |  |  |
| MEBS Total Score |  |  |  |  |
| Total (c^2^) | **.34 (.02, .48)** | **.40 (.30, .50)** | <.00 (.00, .34) | - |
| ACF |  |  |  |  |
| Total (c^2^) | .28 (.00, .47) | **.39 (.29, .49)** | .08 (.00, .42) | - |
| Attributable (c^2^_a_) | .14 (.00, .43) | .04 (.00, .11) | .08 (.00, .42) | - |
| Residual (c^2^_r_) | .14 (.00, .40) | **.35 (.26, .44)** | <.01 (.00, .41) | - |
| % Attributable | 50.0% | 10.3% | 100% | - |
|  |  |  |  |  |
| Non-shared environmentality estimate | |  |  |  |
| MEBS Total Score |  |  |  |  |
| Total (e^2^) | **.57 (.46, .69)** | **.60 (.50, .70)** | **.47 (.34, .63)** | **.47 (.34, .63)** |
| ACF |  |  |  |  |
| Total (e^2^) | **.57 (.45, .69)** | **.61 (.51, .71)** | **.33 (.23, .48)** | **.32 (.23, .46)** |
| Attributable (e^2^_a_) | **.09 (.04, .16)** | **.07 (.03, .13)** | .02 (.00, .07) | .02 (.00, .07) |
| Residual (e^2^_r_) | **.48 (.38, .60)** | **.54 (.45, .63)** | **.31 (.22, .46)** | **.31 (.21, .44)** |
| % Attributable | 15.7% | 11.5% | 6.1% | 6.5% |

*Note.* MEBS= Minnesota Eating Behaviors Survey; ACF= Appearance Conversations with Friends Questionnaire; h^2^= total heritability; h^2^_a_= heritability of ACF that is attributable to genetic effects of MEBS Total Score; h^2^_r_= heritability of ACF that is independent of genetic effects of MEBS Total Score; c^2^= shared environmentality; c^2^_a_= shared environmentality of ACF that is attributable to common shared environmental effects of MEBS Total Score; c^2^_r_= shared environmentality of ACF that is independent of shared environmental effects of MEBS Total Score; e^2^= non-shared environmentality; e^2^_a_= non-shared environmentality of ACF that is attributable to common non-shared environmental effects of MEBS Total Score; e^2^_r_= non-shared environmentality of ACF that is independent of non-shared environmental effects of MEBS Total Score; % Attributable = the percentage of variance attributable to MEBS Total Score out of the total variance underlying ACF

Supplemental Table 10. Parameter Estimates for Bivariate Model for Disordered Eating and the Friends as a Source of Influence Questionnaire

|  | Pre-Pubertal Sample | | Pubertal Sample | |
| --- | --- | --- | --- | --- |
|  | Full Model: ACE | Best Fitting: CE | Full Model: ACE | Best Fitting: AE |
| **Bivariate correlations** |  |  |  |  |
| Additive Genetic (r_a_) | .25 (-1.00, 1.00) | **.44 (.22, .63)** | .99 (-1.00, 1.00) | **.65 (.38, .89)** |
| Shared Environmental (r_c_) | 1.00 (-1.00, 1.00) | **-** | -1.00 (-1.00, 1.00) | - |
| Non-shared Environmental (r_e_) | **.33 (.18, .47)** | **.32 (.17, .45)** | **.34 (.14, .52)** | **.33 (.47, .50)** |
|  |  |  |  |  |
| **Cholesky decomposition models** | |  |  |  |
| Heritability estimate |  |  |  |  |
| MEBS Total Score |  |  |  |  |
| Total (h^2^) | .09 (.00, .46) | **.47 (.35, .57)** | **.51 (.12, .65)** | **.53 (.37, .66)** |
| FSI |  |  |  |  |
| Total (h^2^) | **.39 (.14, .55)** | **.45 (.31, .57)** | .10 (.00, .53) | **.39 (.18, .55)** |
| Attributable (h^2^_a_) | .02 (.00, .54) | **.09 (.02, .19)** | **.10 (.04, .43)** | **.16 (.05, .32)** |
| Residual (h^2^_r_) | .36 (.00, .48) | **.36 (.24, .48)** | <.01 (.00, .36) | **.22 (.05, .38)** |
| % Attributable | 5.1% | 20.0% | 100.0% | 41.0 % |
|  |  |  |  |  |
| Shared environmentality estimate | |  |  |  |
| MEBS Total Score |  |  |  |  |
| Total (c^2^) | **.34 (.02, .50)** | **-** | .01 (.00, .34) | - |
| FSI |  |  |  |  |
| Total (c^2^) | .06 (.00, .24) | **-** | .24 (.00, .44) | - |
| Attributable (c^2^_a_) | **.06 (.01, .24)** | **-** | .24 (.00, .44) | - |
| Residual (c^2^_r_) | .00 (.00, .13) | **-** | <.01 (.00, .32) | - |
| % Attributable | 100% | - | 100% | - |
|  |  |  |  |  |
| Non-shared environmentality estimate | |  |  |  |
| MEBS Total Score |  |  |  |  |
| Total (e^2^) | **.57 (.46, .70)** | **.53 (.43, .65)** | **.47 (.35, .64)** | **.47 (.34, .63)** |
| FSI |  |  |  |  |
| Total (e^2^) | **.55 (.43, .70)** | **.55 (.43, .22)** | **.66 (.47, .84)** | **.61 (.45, .82)** |
| Attributable (e^2^_a_) | **.06 (.02, .13)** | **.05 (.02, .12)** | **.08 (.01, .20)** | **.07 (.01, .18)** |
| Residual (e^2^_r_) | **.49 (.39, .62)** | **.49 (.39, .62)** | **.58 (.41, .74)** | **.54 (.40, .73)** |
| % Attributable | 12.2% | 9.1% | 12.1% | 11.5% |

*Note.* MEBS= Minnesota Eating Behaviors Survey; FSI= Friends as a Source of Influence Questionnaire; h^2^= total heritability; h^2^_a_= heritability of FSI that is attributable to genetic effects of MEBS Total Score; h^2^_r_= heritability of FSI that is independent of genetic effects of MEBS Total Score; c^2^= shared environmentality; c^2^_a_= shared environmentality of FSI that is attributable to common shared environmental effects of MEBS Total Score; c^2^_r_= shared environmentality of FSI that is independent of shared environmental effects of MEBS Total Score; e^2^= non-shared environmentality; e^2^_a_= non-shared environmentality of FSI that is attributable to common non-shared environmental effects of MEBS Total Score; e^2^_r_= non-shared environmentality of FSI that is independent of non-shared environmental effects of MEBS Total Score; % Attributable = the percentage of variance attributable to MEBS Total Score out of the total variance underlying FSI.

Supplemental Table 11. Parameter Estimates for Bivariate Model for Disordered Eating and the Peer Attribution Scale

|  | Pre-Pubertal Sample | | Pubertal Sample | |
| --- | --- | --- | --- | --- |
|  | Full Model: ACE | Best Fitting: CE | Full Model: ACE | Best Fitting: AE |
| **Bivariate correlations** |  |  |  |  |
| Additive Genetic (r_a_) | -.81 (-1.00, 1.00) | - | **.43 (.04, 1.00)** | **.43 (.16, .66)** |
| Shared Environmental (r_c_) | **.98 (.26, 1.00)** | **.46 (.27, .64)** | -1.00 (-1.00, 1.00) | - |
| Non-shared Environmental (r_e_) | **.34 (.19, .46)** | **.28 (.16, .39)** | **.25 (.03, .44)** | **.25 (.03, .44)** |
|  |  |  |  |  |
| **Cholesky decomposition models** | |  |  |  |
| Heritability estimate |  |  |  |  |
| MEBS Total Score |  |  |  |  |
| Total (h^2^) | .10 (.00, .44) | - | **.53 (.08, .66)** | **.53 (.37, .66)** |
| PAS |  |  |  |  |
| Total (h^2^) | .20 (.00, .50) | - | **.51 (.22, .66)** | **.51 (.31, .66)** |
| Attributable (h^2^_a_) | .13 (.00, .48) | - | .09 (.00, .56) | **.09 (.01, .24)** |
| Residual (h^2^_r_) | .07 (.00, .48) | - | .41 (.00, .56) | **.41 (.24, .56)** |
| % Attributable | 65.0% | - | 17.6% | 17.6 % |
|  |  |  |  |  |
| Shared environmentality estimate | |  |  |  |
| MEBS Total Score |  |  |  |  |
| Total (c^2^) | **.32 (.05, .48)** | **.40 (.30, .50)** | <.01 (.00, .38) | - |
| PAS |  |  |  |  |
| Total (c^2^) | **.27 (.02, .49)** | **.44 (.33, .43)** | <.01 (.00, .20) | - |
| Attributable (c^2^_a_) | **.26 (.01, .48)** | **.09 (.03, .19)** | <.01 (.00, .20) | - |
| Residual (c^2^_r_) | .01 (.00, .37) | **.34 (.24, .44)** | <.01 (.00, .20) | - |
| % Attributable | 96.3% | 20.5% | 100% | - |
|  |  |  |  |  |
| Non-shared environmentality estimate | |  |  |  |
| MEBS Total Score |  |  |  |  |
| Total (e^2^) | **.57 (.46, .69)** | **.60 (.50, .70)** | **.47 (.34, .64)** | **.47 (.34, .63)** |
| PAS |  |  |  |  |
| Total (e^2^) | **.53 (.42, .65)** | **.56 (.47, .67)** | **.49 (.34, .69)** | **.49 (.34, .69)** |
| Attributable (e^2^_a_) | **.06 (.02, .12)** | **.04 (.01, .09)** | .03 (.00, .11) | .03 (.00, .11) |
| Residual (e^2^_r_) | **.47 (.37, .59)** | **.52 (.43, .62)** | **.46 (.33, .64)** | **.46 (.33, .64)** |
| % Attributable | 11.3% | 7.1% | 6.5% | 6.1% |

*Note.* MEBS= Minnesota Eating Behaviors Survey; PAS= Peer Attribution Scale; h^2^= total heritability; h^2^_a_= heritability of PAS that is attributable to genetic effects of MEBS Total Score; h^2^_r_= heritability of PAS that is independent of genetic effects of MEBS Total Score; c^2^= shared environmentality; c^2^_a_= shared environmentality of PAS that is attributable to common shared environmental effects of MEBS Total Score; c^2^_r_= shared environmentality of PAS that is independent of shared environmental effects of MEBS Total Score; e^2^= non-shared environmentality; e^2^_a_= non-shared environmentality of PAS that is attributable to common non-shared environmental effects of MEBS Total Score; e^2^_r_= non-shared environmentality of PAS that is independent of non-shared environmental effects of MEBS Total Score; % Attributable = the percentage of variance attributable to MEBS Total Score out of the total variance underlying PAS.


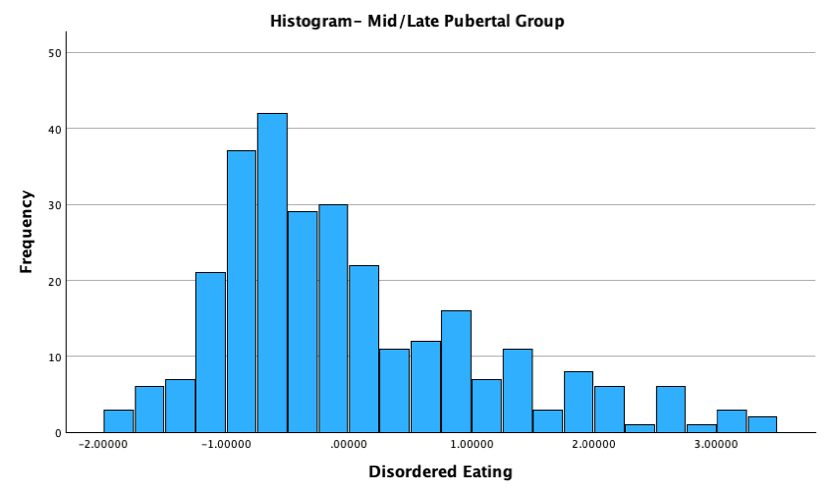

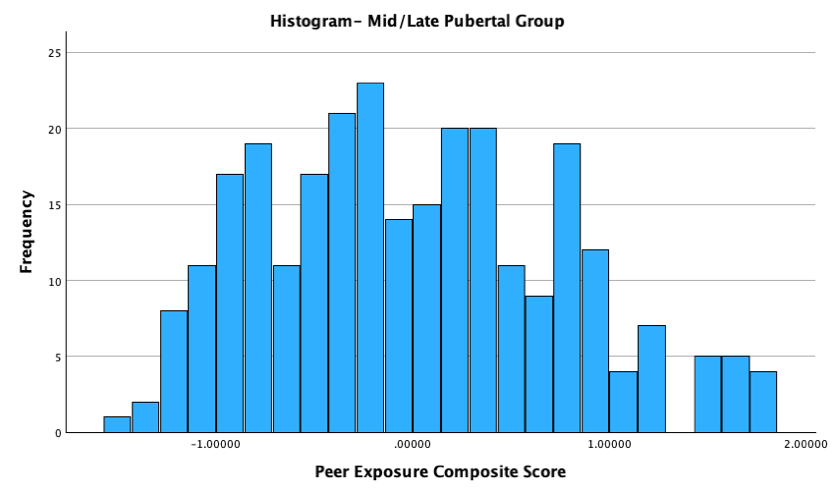

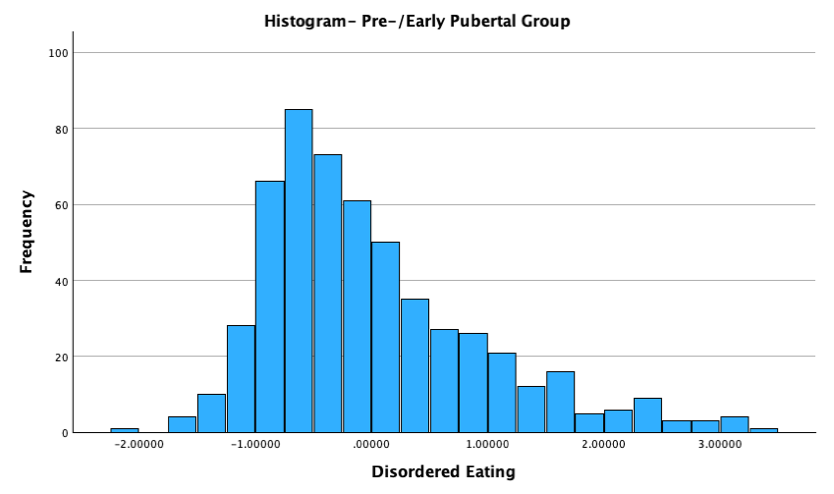

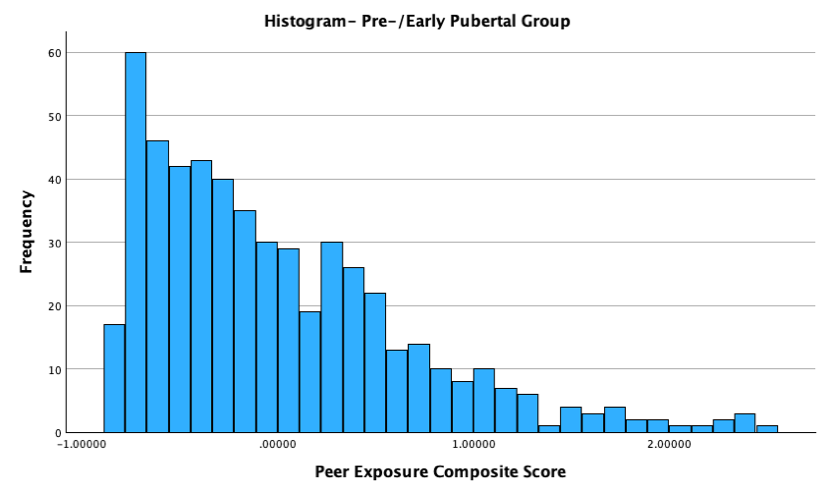

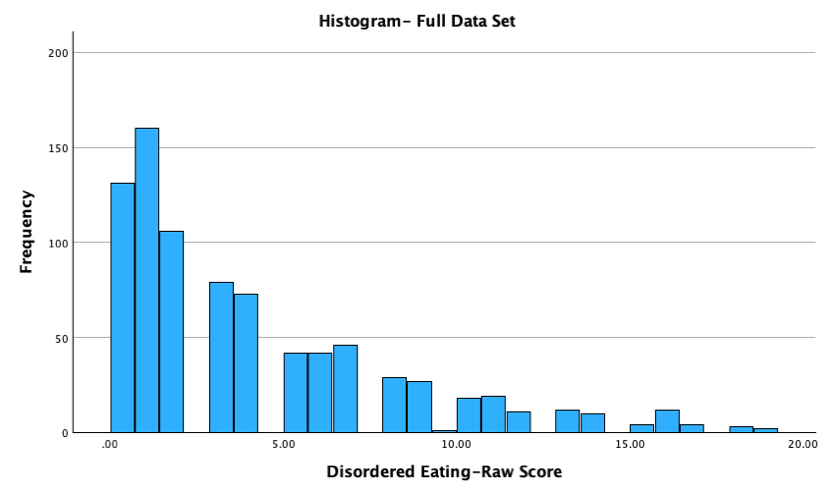

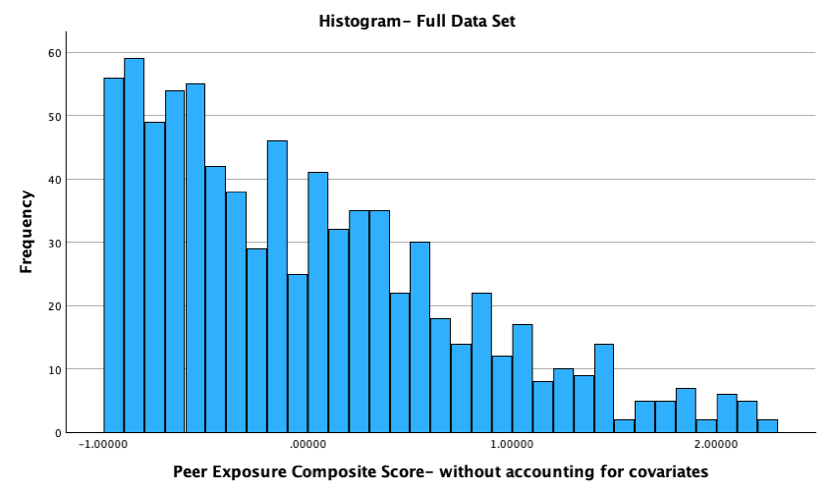


*Supplemental Figure 1*. Histograms are provided for the raw variables using the full data set (top row). Histograms of the analytic variables in the pre-/early pubertal group (middle row) and mid-/late pubertal group (bottom row) are also provided.

**Appendix A: Additional Information on the TSMBH Study**

The TSMBH recruited twins from the Michigan Twins Project (MTP), a population-based recruitment database that recruits twins ages 3-55 years using birth records in collaboration with the Michigan Department of Health and Human Services (see Burt & Klump (2013) for recruitment details). The response rate (57%) for the MTP is on par or better than that of other twin registries that use similar recruitment methods (Iacono & McGue, 2002), and response rates for the TSMBH were even higher at 66%.

The TSMBH study investigated the influence of ovarian hormones on phenotypic and genetic risk for DE during puberty. Thus, several inclusion/exclusion criteria were applied to ensure accurate hormone sampling (e.g., no hormonal conceptive in past 3 months, no psychotropic, steroid, or hormone-related medications in the past 4 weeks, no pregnancy or lactation in the past 6 months, no genetic or medical condition known to influence hormone functioning, appetite or weight). Notably, however, TSMBH twins were not significantly different from non-participating MTP families in overall DE symptoms (assessed using the MTP ED questionnaire composite score; *t*(391)=-0.95 *p*=0.35) or BMI (*t*(375)= -0.84 *p*=0.40). The recruited TSMBH was also highly representative of the MTP sample and the general population of Michigan in terms of ethnic/racial distributions, with 4.4% of pairs identifying as Hispanic and 81.5% identifying as White, 8.3% as African American, 0.2% as American Indian/Alaskan Native, and 9.3% as multi-racial.
